# Evaluating barriers to reaching women with public health information in remote communities in Mali

**DOI:** 10.1186/s12913-024-11277-5

**Published:** 2024-08-07

**Authors:** Robert T. Jones, Freya I. Spencer, Laura A. Paris, Diarra Soumaïla, Nanthilde Kamara, Alexandra Hiscox, James G. Logan

**Affiliations:** 1 Arctech Innovation, LondonEast-UK business and technical park, Dagenham, UK; 2 Department of Disease Control, London School of Hygiene & Tropical Medicine, Keppel Street, London, UK; 3 Data Blon consulting, District de Bamako, Mali; 4 Viamo, Bamako, Mali

**Keywords:** Health information, Women, Remote communities, Mali, Barriers to access, Mobile communication

## Abstract

Telecommunications offers an alternative or supplement to community-based interventions as a means of extending healthcare services and improving health outcomes in remote settings but can fail to reach target communities and achieve the desired impact if barriers to access are not overcome. We conducted seven focus group discussions and 26 interviews with community health workers, community leaders, and female members of the public who declared that they had or had not previously accessed free audio health messages provided via a mobile platform in two rural communities of Mali, Koulikoro and Bougouni. A content analysis showed that participants accessed and trusted health information from a range of sources, including radio, telephone and television, as well as town criers, local relays and community health centres. Barriers to access faced by women included economic factors, lack of network or electricity, and social factors such as illiteracy, cultural restrictions and being unaware of mobile communication. Through analysis and interpretation of the participants’ responses, we have made recommendations for future campaigns for the dissemination of health-related information for women in remote settings.

**Supplementary Information:**

The online version contains supplementary material available at 10.1186/s12913-024-11277-5.

## Introduction

Community-based interventions offer a means of extending healthcare services and improving health outcomes, particularly in rural settings. The mainstays of these initiatives in Africa are community health workers (CHWs), trained lay people who live in the communities they serve and who provide a critical link with the primary healthcare system [1]. With appropriate support, CHWs are able to provide lifesaving prevention and treatment services for many conditions, direct patients and their families to appropriate sources of care, and build trust health services [2].

CHWs are well positioned to provide health-related information to those in their communities, but there are limitations to their effectiveness. Contextual, socioecological factors affect the engagement of CHWs, who may have limited training and lack the knowledge necessary to effectively perform their responsibilities [3, 4]. These health workers may also face challenges balancing their duties with other workloads, difficulties accessing patients’ homes, or a lack of the resources needed to assist their communities [5, 6]. There are also barriers to patients accessing the services provided by CHWs. For example, a study in Malawi reported that individuals with HIV and tuberculosis experienced stigma and discrimination in their communities because CHW visits were associated with the presence of an HIV patient [7].

We previously employed a telephone-based messaging campaign (which we refer to as the ‘3-2-1 service’) to reach individuals in remote communities of Mali with a series of messages promoting health-seeking behaviours related to malaria and COVID-19. [8]. Approximately 130,000 unique listeners accessed the service every month, dialling a free-phone number (3–2–1) to listen to pre-recorded messages. However, review of listener data found that only 17% of those accessing the messaging service were calling from phone numbers that were registered to women, suggesting that women did not receive the full benefits of accessing information that could help protect them and those in their care.

It is estimated that approximately 90% of people in Mali own a mobile phone and that ownership is slightly greater in rural areas than in urban areas; therefore, there are opportunities to use mobile communication to address health issues [9, 10]. Mobile phones can be used for phone surveys, calls or Short Message Service (SMS)/text messages between clients and health practitioners, such as to remind people to attend medical appointments and take prescribed medication [11, 12]. Informational messages can be developed for caregivers that provide guidance on disease prevention and illness management or that can encourage health seeking behaviours [13, 14]. Indeed, the use of mobile wireless technologies for health, known as mobile health or mHealth, offers unique opportunities for service delivery. Whilst the innovative role that digital technologies can play in strengthening health systems are recognised, there is an important need to evaluate their contributing effects and ensure that investment in such programs do not inappropriately divert resources from alternative, non-digital approaches [15]. At present, high quality evidence on the effectiveness of such interventions remains limited [16].

Women are often the primary caregivers of their families and are responsible for the nutrition, health and well-being of their children and families. A recent scoping review identified poor access and utilisation of healthcare information by women of reproductive age in low- and middle-income countries and called for more primary studies to determine the accessibility, financial accessibility, connectivity, and challenges faced by women in these settings [17]. In this study, we employed in-depth interviews to investigate the reasons why women accessed or did not access a mobile messaging service in Mali. We also used in-depth interviews and focus group discussions with health workers and community leaders to explore barriers that might be faced by people trying to access health-related information and alternative means of reaching vulnerable populations.

## Methods

### Study setting

Participants in this study were recruited from rural communities in two Malian regions—Koulikoro, in the health district of Kati (region of Koulikoro), and Bougouni in the health district of Ouélessébougou (region of Sikasso). These regions have mean estimated International Wealth Index (IWI) [18] values ranging from 44.2 (Koulikoro) to 45.1 (Sikasso), compared to 68.8 in the capital region, Bamako [19]. The population densities in Koulikoro and Sikasso are 31.9 and 47.5 people per km^2^, respectively. Bamanankan is the most widely spoken language in Mali.

The Koulikoro region is Mali’s 2nd largest administrative region, bordering Guinea and Mauritania. It is a major area of agricultural production (cereals and peanut cotton) and livestock farming. There are several gold-panning sites in the region. The population is predominantly Bambara, Malinké and Peulh, all of whom are sedentary.

The Sikasso es region is Mali’s 3rd administrative region and borders Côte d’Ivoire. It is highly suitable for agriculture, and is the country’s second-largest cereal-producing area and leading cotton-producing region. It is also the country’s leading fruit (mango and citrus) and vegetable-producing region. Due to insecurity in the northern regions, and the south boasting abundant pasture and water for water animals, many breeders have migrated to the Sikasso region with their herds. Several gold mines are being exploited in the region, and industrial lithium and diamond infrastructures are under construction. There are also several gold panning sites. The region is mainly populated by Bambara, Sénoufo, Minianka and Peulh, all of whom are sedentary.

### Individual interviews

**Fig. 1:**
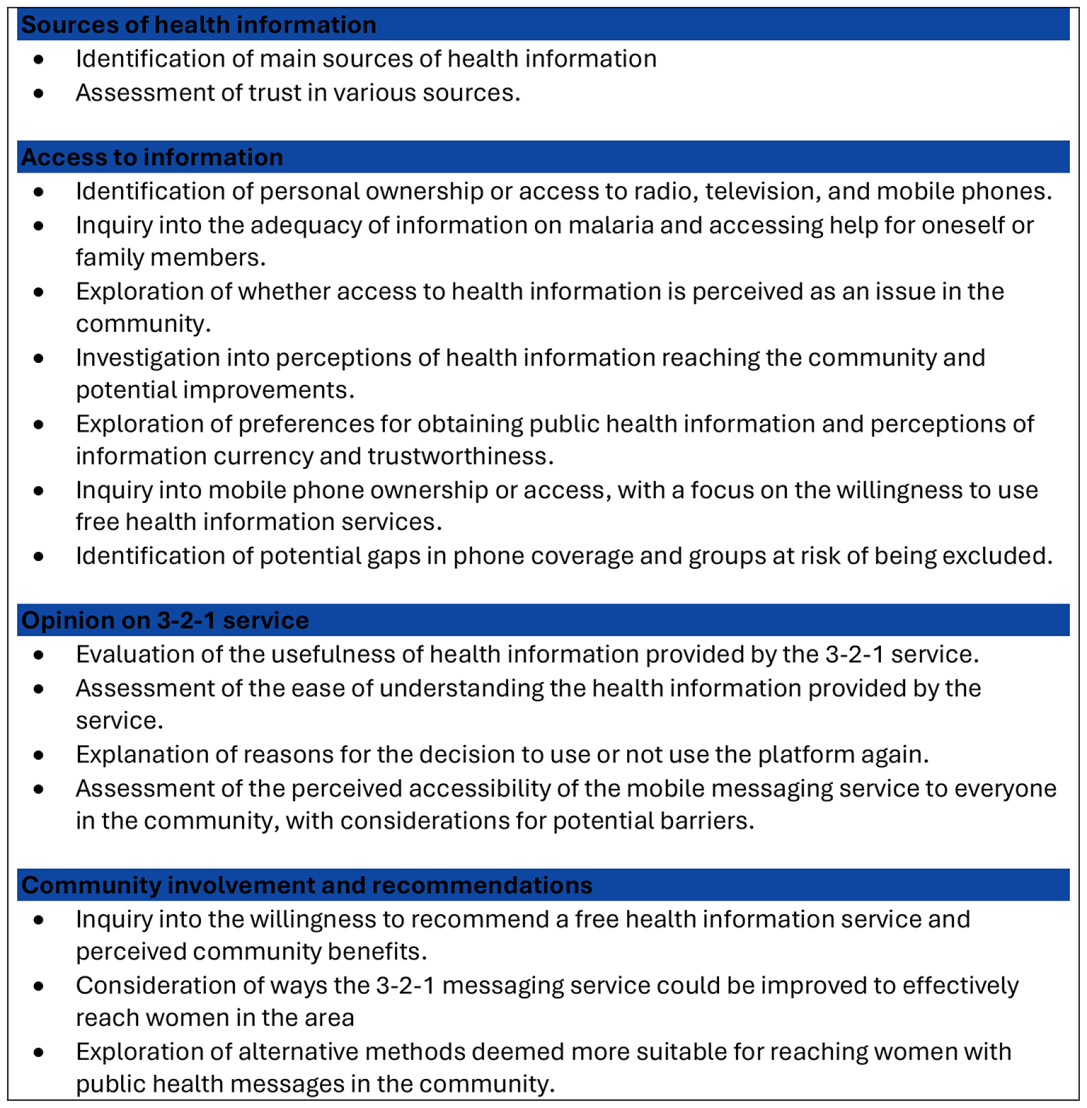
Discussion themes of interviews and focus group sessions

In-depth interviews were held with 26 women living in areas previously targeted by the mobile messaging service. Participants were eligible if they were aged 18 years or older, self-identified as women, resided in a rural area of Mali previously targeted by the 3-2-1 service and were able to confirm that they did or did not engage with the 3-2-1 service in the past. Purposive sampling was used to select individuals who had previously engaged with the 3-2-1 service and those who had not. The interviews were also held with health workers. The themes of the sessions are shown in Fig. 1, and an interview guide is provided in the Supplementary Material. The question guide was developed following a preassessment and review of the protocol by the Ministry of Health. The interviews were conducted in French, a language spoken by all participants. On occasion, Dogon was spoken by individuals and so a collective agent who could speak Dogon, was present.

## Focus group discussions

Seven focus group discussions were conducted with women who had not previously accessed the 3-2-1 service, CHWs and community leaders. CHWs of any gender were eligible if they had operated within rural communities for a period of more than 6 months. Community leaders were considered eligible if they were religious leaders, governmental officials or community/tribal chiefs. These groups were selected to obtain a well-rounded view of the challenges faced in rural settings and to obtain informed input from key groups within these communities in Mali. The question guides used in the sessions followed the same themes as those used in the in-depth interviews (Fig. 1 and Supplementary Material). Focus group discussions were held in French, a language spoken by all participants.

### Participant recruitment

Written informed consent was obtained from all participants. All interview and focus group discussions were recorded using an audio recorder and transcribed by a member of the field team. The transcripts were translated into English prior to analysis. The sessions took place in August and September 2023.

### Thematic analysis

Open coding was performed in NVivo (V.14, QSR International). Theme generation followed Braun and Clarke’s six phases for thematic analysis [20]. A preliminary coding framework was established from the topic guide, but coding was mostly inductive by grouping prevalent response patterns into higher-order categories. The 32-item Consolidated Criteria for Reporting Qualitative Research tool was used to ensure that all key methodological issues were considered (COREQ checklist provided in the Supplementary Material).

## Results

A total of 26 in-depth interviews were held (Table 1). The age of the female participants who had previously accessed the 3-2-1 service ranged from 18 to 48 years. The age of the females who had not previously accessed the 3-2-1 service ranged from 18 to 35 years. The community health workers included four males and five females, with occupations including patient consultant, midwife, community health agent, vaccinating agent, director of the health centre and paediatric nurse. Seven focus group discussions were held and included a total of 56 participants, 32 of whom were female (Table 1). For brevity, quotes are coded: H = Health worker, L = Community leader, U = 3-2-1 User, NU = 3-2-1- Nonuser, I = Interview, FG = Focus group, followed by the session number. For example, Koulikoro-U-I-1 refers to interview session 1 held with a 3-2-1 service user in Koulikoro.

**Table 1:** Number of participants by session type and region

Type of session	Participants	Koulikoro	Bougouni	Total number of participants	Number of women
In-depth Interviews (*N* = 26)	Community health workers	5	4	9	5 (56%)
	Women who have previously accessed 3-2-1 service	5	4	9	9 (100%)
	Women who have not previously accessed 3-2-1 service	4	4	8	8 (100%)
Focus groups (*N* = 7)	Community leaders	10	8	18	0 (0%)
	Community health workers	7	13	20	14 (70%)
	Women who have not previously accessed 3-2-1 service	10	8	18	18 (100%)

### Thematic analysis

**Fig. 2:**
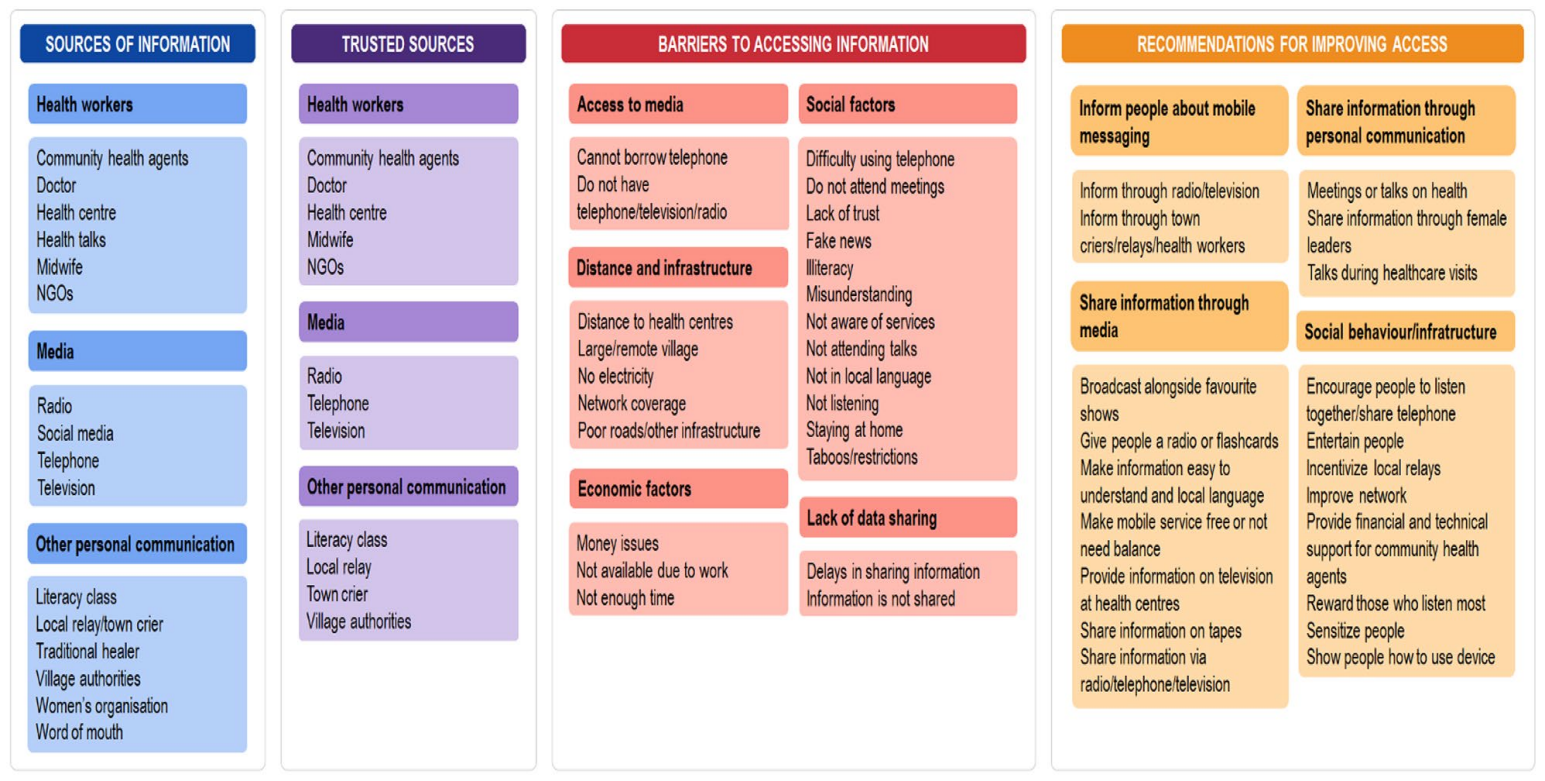
**Themes of the central research questions.** (1) Which sources provide health information? (2) Which sources of health information do you trust? (3) What are the barriers to accessing health information? (4) What recommendations do you have for improving access to health information?

Responses to the interview transcripts were coded into the central themes of sources of health information, trust in these sources, barriers to accessing health information, and recommendations for improving access to health information (Fig. 2). Responses to questions exploring reasons for accessing or not accessing the 3-2-1 service and whether the service was useful were also coded.

### Sources of information

Participants described a range of sources of health information, which may be categorised as (1) health workers, (2) media, or (3) other personal communication (Fig. 2). Information from health workers was delivered via educational talks led by health workers or NGO agents during vaccination campaigns and prenatal visits or during consultations with health personnel. Community health workers or agents were also cited as a source of information disseminated during home visits, during women’s gatherings or upon request. The community health platform is a term used to describe any community service based in the village. It provides structure for CHWs as well as the service for community members (community relays, GSAN, mother and father educators), and was described as a focal point for health activities in the village, such as vaccination programmes or community nutrition support groups (known locally as ‘GSANs’).

Community radio stations were described as providing health shows, and television was also a frequent source of information for some participants. Participants also mentioned the telephone, including the Viamo 3-2-1 platform, messages from Orange Mali and its partners, and social networks. Like Viamo, Orange Mali can share information by SMS and has a programme called Senekela that is primarily aimed at providing farmers with information to improve their agricultural practices. The sources of information varied across participants, with some clearly preferring some sources and not utilising or valuing others. Community relays are typically a designated man and a women within a community, who facilitate health actions such as vaccination, mass distribution of medicines and referrals of patients to a higher level. Those in very remote settings said that community relays or nurses do not visit them and that they have no television or radio. For them, the telephone is the main source of information.

*“I get [information] from my telephone. We don’t have a television. That’s why we are using a phone to listen to the service*” (Koulikoro-U-I-1).

*“Telephone is my source of health information. Telephone is more practical*. (Bougouni-U-I-3.)

*“Local relays share information they get with all the community members. Few people listen to the radio. Few people have television sets*,* too. So*,* local relays remain the principal source of information for the community*” (Koulikoro-L-FG-2).

*“People get information about health from the traditional town criers who use drums to share the information. Also*,* some people get information from the local relays and radio*,* and others from television”* (Koulikoro-H-FG-1).

### Trust in information sources

Trust was discussed with reference to sources of health-related information. Participant responses indicated that they had confidence in information coming from the three categories mentioned above (health workers, media, and other personal communication). Many participants trusted information received directly from people working in the health sector, including doctors, community health agents and other health personnel. Other participants indicated that there was trust in people known within the community, but this opinion was shared more by community leaders and those working in the health sector.

*“In this rural community*,* people trust information delivered from health agents directly. Villagers believe more in the health agents than [information] shared on social networks. Also*,* they believe in what the town criers share because it comes from the village authorities”* (Koulikoro-H-FG-1).

*“I trust doctors. You feel healthy when you listen to doctors” (Bougouni-NU-I-4).*

*“They believe in the community health centre agents because the agents are in contact with them. They know how to sensitize them. It is the health agents who treat them when they get sick. Many villagers are healthy because of them. Health is their job”* (Koulikoro-H-FG-1).

Some members of communities expressed that they found television and radio to be reliable sources, and for some, this was because the information they shared came from doctors or was widely shared.

*“We believe in radio because their information comes from doctors. We believe in our local doctors”* (Bougouni-L-FG-1).

*“I believe in the health information shared on the radio by doctors*” (Bougouni-H-FG-1).

Despite the range of trusted sources, some participants indicated that some may be less reliable than others and, in particular, focused on false information spread on social networks. There was evidence of misinformation in some of the responses given by the participants. When asked what she knew about malaria, one participant stated:

*“I know that when you eat a meal with much oil*,* cucumber*,* or watermelon a lot*,* you can catch it”* (Koulikoro-NU-I-4).

*“They must trust the health information of the community health centres. But*,* they also trust other sources like fake news on social media on health information”* (Koulikoro-H-I-4).

*“Some rumours on social media and some influential people giving wrong information to the community members. They frighten them*,* telling them they will die or get ill if they do this treatment… That’s why some people reject vaccination”* (Koulikoro-H-I-4).

### Usefulness of mobile messaging services

Although one person said that it is easier to access information on the radio (Bougouni-H-I-2), all those who were aware of the 3-2-1 service and had used it found that the health information provided was useful. Content was provided in their local language rather than in French and was described as having a large amount of relevant health information. Some also indicated that they had experienced a change in behaviour following listening to the messages. A point made in the interviews was that messaging via mobile phones gives people the flexibility to listen to information when they have time.

*“I have learned about the symptoms of malaria. People must go to the health centre to treat it. We used to treat it at the traditional healers*,* but today more people go to the health centre”* (Koulikoro-U-I-3).

*“Sharing health information on phones is more accessible to people than other ways… You can call the service anytime for health information with phones. But*,* on televisions and radios*,* there is a time set for health information. You can miss it sometime”* (Bougouni-L-FG-1).

### Reasons for not accessing the 3-2-1 service

The majority of participants who had not previously accessed the 3-2-1 service stated that there were not aware of its availability. However, lack of mobile network coverage was mentioned by some participants, principally by those who indicated that they live in a particularly remote region. Others also stated that they do not have a telephone or that their phone was broken. Three participants also mentioned that they do not have electricity.

*“We don’t have electricity here. Many people don’t know about the service here”* (Koulikoro-U-I-3).

*“I don’t think network coverage will be an obstacle. There is network coverage almost everywhere today. In the area of Ouelessebougou*,* about 44 villages*,* there is network coverage”* (Bougouni-H-I-1).

Although the 3-2-1 service is available for free, a minimum balance of at least 100 West African CFA franc (approximately $0.16) is required on the mobile telephone in order to access the 3-2-1 messages. This restriction was also mentioned by several participants, as was confusion about whether the service consumed mobile credit.

*“The health information on Viamo [3-2-1] service is essential for people in remote places who don’t have access to information. In those places*,* people live in poor conditions. They can’t afford a phone balance. They can’t have access to balance in some villages”* (Koulikoro-H-I-4).

*“Many people will call the service when it is free because they don’t always have money to buy a balance. Because some people can’t access the service when they are out of balance three days later*,* they stop calling it. They think it is consuming”* (Bougouni-U-I-4).

### Access to health information

The interview and focus group sessions provided insight into obstacles to disseminating health-related information within the participants’ communities. Responses fell into the categories of (1) access to media, (2) distance and infrastructure, (3) economic factors, (4) social factors, and (5) lack of data sharing (Fig. 2). Young people were frequently identified as groups that would have less access to information because they have less autonomy and do not have a radio, telephone or television, but women and working men were also mentioned, as were elderly people who may stay at home and not travel to health centres. When asked what group of people do not have phones and who might be missed by information shared by mobile messaging, they responded:

*“Females*,* because they can’t keep a phone”* (Bougouni-H-I-4).

*“Many young people don’t get health information because they don’t have phones*” (Bougouni-U-I-1).

These views about limited access to phones were not held by all and were contradicted by a number of individuals in interviews and focus groups who said that mobile phones were a source of information. Indeed, one participant confirmed:

*“Most of the women here have phones”* (Bougouni-NU-I-1).

​ Economic factors were raised, with many participants stating that issues with insufficient time or money prevented them from receiving health information.

*“Lack of money is the principal reason. They all say they go to the health centre late because of money issues to buy the medicaments… When he can’t afford it*,* they go to traditional healers”* (Bougouni-H-I-4).

*“Some farmers don’t access health information. They are always busy working on farms”* (Bougouni-H-FG-1).

Religious and cultural factors were discussed as potential obstacles to accessing health-related information, particularly the control of information by husbands. One health worker also indicated that men are overlooked as targets for health information.

*“[Men] aren’t targets of the health policy. We work with women a lot in the field of health*” (Bougouni-H-I-1).

*“Some husbands don’t support their wives in getting health information…Some husbands don’t want their wives to listen to family planning information because it is taboo. They think that family planning is against the teaching of religions”* (Koulikoro-H-I-4).

*“Some elders don’t let young people listen to some health issues”* (Bougouni-H-I-3).

Finally, some indicated that it was not access to information that was the problem but rather to people who were not listening or who misunderstood.

*“Accessing health information is not a challenge*,* but understanding is. The community members believe in the health information from people they know. They won’t trust a stranger”* (Koulikoro-H-I-2).

### Recommendations for improving access to health information

The interviews and focus group discussions provided an opportunity to explore, with target communities as well as their leaders and those working in the health sector, how future efforts could be focused on improving access to health information (Fig. 2).

The interviewers guided participants to respond to the question of how to improve uptake of the 3-2-1 messaging service, and responses typically focused on the need to let people know about it or sensitise them to its importance.

*“You should teach people how to call the service. They don’t know how it works. They will use it if they know how it works because more people have telephones*” (Bougouni-L-FG-1).

*“I suggest informing people about the service on TV and the radio. Many people don’t know about the service. As health workers*,* we should be the first to know about it*,* but many don’t”* (Bougouni-H-I-2).

*“Many women listen to “Baroni” [radio show]. They are very interested in it. It is amusing for them. You can also broadcast the Service health information before “Baroni” starts”* (Bougouni-H-I-1).

Due to the possibility of fake news or misinformation, participants indicated that it is important that mobile communication receive the support of local health agents. This involvement of local and trusted individuals was expressed for information sharing more broadly, not just for informing people about mobile messaging services.

*“Radio broadcast information only. They don’t get in touch with villagers. So*,* villagers cannot believe them more than the health agents”* (Koulikoro-H-FG-1).

*“You need to spread the information about the service so that many people can learn about it. Meet them and talk to them like you are doing it now. When ten people know about it*,* they will inform many people”* (Koulikoro-L-FG-1).

*“I am a vaccination agent. I go from village to village for vaccination campaigns. Before I go*,* local relays tell the community about my arrival*,* but some families tell me that they haven’t got the information about it. Local relays should work hard to share health information with all the community members. They should go to mosques and everywhere to inform the community”* (Bougouni-H-FG-1).

Many participants indicated that they or others in their community like to access information on phones and would dial in if there was not a need for a balance. Some said that when they do not have a balance, they will miss the information, and others were concerned that the service gradually used their credit.

*“I advise sharing health information on phones. They are efficient tools for sharing health information. You can move with them and use them everywhere”* (Bougouni-NU-FG-1).

*“Many people will call the service if CFA 100 is not a requirement. Before I lost my phone*,* I used to listen to it with a balance of CFA 100. But I couldn’t three days later without a balance in my account”* (Koulikoro-U-I-3).

*“You should negotiate with your mobile phone operator partner to make it free from charges for villagers. They can’t afford it”* (Koulikoro-H-FG-1).

Telephones were considered personal devices, and few thought it was practical to borrow a device from someone other than their husband. Instead, they suggested sharing through other sources of media or personal communication. Finally, there were greater structural issues that some participants indicated must be addressed to improve access to information, including addressing illiteracy and network coverage.

*“Women are more interested in entertainment and fun activities. You can attract them by organizing fun activities that incorporate health information.* Entertainment or amusement when broadcasting the information about the service can help draw the attention of many women” (Koulikoro-L-FG-1).

*“I suggest radio or television. Those who don’t have a phone can go and watch television or listen to the radio to people possessing them. Telephones are personal devices*,* but radio and television are not in Siby”* (Koulikoro-L-FG-1).

*“There is a high illiteracy rate here. I receive many messages myself*,* but I can’t read them. Women need to learn how to read and write to use the service”* (Bougouni-L-FG-1).

## Discussion

The use of mobile phones to distribute public health messages represents an exciting opportunity for educating and helping the public to ensure that they are taking appropriate steps to protect themselves and those in their care. Advantages in remote settings, such as those investigated in this study, are that the messages can reach people with low literacy, who may be far from traditional health services, and who have limited economic resources [21, 22]. However, it remains a challenge to reach both men and women equally using this innovative approach [23]. Analysis of data gathered from our previous mobile messaging campaign in Mali indicated that a large proportion of handsets used to access messages were registered to men, although the data gathered did not make it possible to determine whether females might have called the messaging service on a male-registered device. A 2015 survey indicated that ownership of at least one mobile phone in Mali is marginally greater in females (74%) than in males (72%) [10], and our more recent work in Malawi showed a 50:50 male: female ratio when users of the 3-2-1 service were asked to self-report their gender (A. Hiscox, personal communication).

Previous research has shown that mobile communication can be a valuable tool for supporting disease control and has an impact on adherence to treatments [24–26]. A study in Ghana reported a six-fold increase in uptake at a drop-in centre one month after the launch of a hotline for answering questions and encouraging users to seek care [27], and in Uganda, the number of people taking an HIV test doubled the week after a test campaign to 8,000 users began [28]. Methods to determine whether participants have gained knowledge via engagement with an mHealth programme include surveys and text-based quizzes, but knowledge does not necessarily translate into altered habits, and methods to determine whether users have followed guidance, such as to wash their hands, take a drug treatment or conduct a self-breast examination, are typically based on self-reported data [29, 30]. It can be much more challenging to independently assess changes in behaviour, so these reports from Ghana and Uganda, and others of increased attendance at health services are encouraging [31].

However, more evaluations of current interventions need to be conducted to strengthen the evidence base for the value of mHealth services, and there is particularly weak evidence concerning scalability and sustainability [32, 33]. In addition, despite the number of mHealth studies reported, there remains insufficient investigation into best strategies for engagement [34]. Prior to scaling-up initiatives from pilot studies, it is important to understand how best to reach users, maximize engagement and avoid attrition.

Participants in Mali explained that they currently access information through literacy classes, women’s organisations and other gatherings. To promote the health of women, community mobilisation through group activities has been shown to be effective in a range of low and middle-income settings [35]. Mobilisation activities require group participation, so when attendance is poor, fewer community members will be exposed to new information or behaviour change communication. Previous focus groups have identified a range of factors that might prevent people from attending community engagement events, such as the need to prioritise farming activities [36], and the responses people gave in our study relating to time pressures suggest that similar issues would be faced in Mali. Although informal information sharing is expected to occur, when group members pass on to others health information they have received, particularly in more rural areas with greater social cohesion [37, 38], social structures and power dynamics could affect this communication. It is important that women are reached directly to ensure fidelity in the messages they receive [39].

Tools to disseminate public health information or improve health-seeking behaviours directly include the adoption of radio education, TV and newspaper advertisements, talk shows and documentary series, live concerts, and musical shows [40–42]. Interviews held in other sub-Saharan Africa countries have previously found participants accessing a range of sources for health information and identified infrastructural barriers such as poor roads and lack of electricity [43, 44]. While some participants in our study indicated that mobile network coverage remained an issue in their community, others disagreed and did not consider this to be a barrier to mobile communication. Instead, a cultural barrier to information access was described: several participants indicated that certain health subjects were not discussed with particular groups. This challenge has been described previously from studies in Mali [45], and raises the importance of sensitisation and the need to overcome taboos or cultural restrictions that might prevent individuals from receiving information that is important to their health.

Town criers or relays were cited as a source of trusted information by community leaders, health workers and members of the community, because they were known to people in their locality. Voluntary community relays have become important components of African community health policy, but previous research in Mali has suggested that people carrying out the role of relays encountered difficulties in devoting sufficient time to this activity and had to make economic compromises through a lack of remuneration [46]. Challenges related to illiteracy have also been discussed as limitations to their impact, despite a requirement for community relays to be literate [47]. Furthermore, it has been suggested that nonrenewal of volunteer agents seriously threatens the longevity of the system [46], implying that community relays might not be a sustainable source of information sharing in the future.

Based on the findings of the content analysis, our recommendation for a future health information campaign is to utilise a mobile messaging platform such as the 3-2-1 service that is advertised alongside popular local radio shows as well as promoted locally by health workers, town criers, relays and local authorities to inform those who might otherwise be unaware. The trust of communities in doctors and health workers should also be leveraged. At face-to-face appointments, doctors, nurses, midwives, other health professionals or CHWs could inform people about the service and encourage them to use it, particularly with their partners or in groups, and then those individuals would have access to health information at intervening times. The use of telecoms for communication of health information could be particularly beneficial for taboo topics where relays or other forms of face-to-face communication might not reach the desired audience. Opportunities to incentivise or reimburse health professionals and CHWs should be considered, as well as opportunities to raise awareness with them at routine training or requalification sessions and any programmes that they might be involved with at NGOs or as part of academic research projects. The messaging service can make the jobs of health professionals easier by providing health information and encouraging health-seeking behaviour before illness becomes too severe. Finally, it is important that the messages be broadcast in regional languages, that they receive approval from the Ministry of Health or local authorities, and that they align with the advice of local doctors. Content should be easy to understand but also entertaining, to the extent that it can be listened to in women’s groups, literacy classes and other gatherings.

Our vision is to improve the lives and health of underserved communities around the world. Our recommended programme, which makes use of both a mobile messaging platform and personal contact with known and trusted individuals, could immediately be rolled out across sub-Saharan Africa, where malaria, HIV, tuberculosis and other diseases cause hundreds of thousands of deaths and widespread suffering in rural communities [48, 49], and then adapted for other regions. When literacy is higher, the incorporation of chatbot features could improve accessibility and education [50] and support referrals to local clinics and reminders to take medication for those managing chronic diseases. Expansion may allow for features similar to those offered by the NHS 111 in the United Kingdom or HealthDirect Australia [51, 52], further extending the reach of the health system and reducing pressure on local services.

### Study limitations

The interviews were led by a consultant engaged by Viamo Mali, whose line of questioning or undocumented emphasis may have influenced the responses of some participants. Furthermore, a considerable proportion of participants were community leaders and health workers whose opinions were valuable but may have been biased and altered the balance of responses given. All community leaders were male, representative of the gender of leaders in the study area, but their views on barriers to reaching women may not have represented the views of women, particularly for taboo topics.

## Conclusion

Our study identified that participants access and trust a range of sources of information, including local health workers and the media. However, economic and social factors continue to limit the ability of women in remote communities to receive information relevant to their health. We recommend that future campaigns are built on mobile communication, but leverage personal contact with local trusted sources and promotion through popular radio programmes to extend healthcare services and improve health outcomes in remote areas.

### Electronic supplementary material

Below is the link to the electronic supplementary material.

Supplementary Material 1

Supplementary Material 2

## Data Availability

All data generated or analysed during this study is included in this published article and its supplementary information files.

## Declarations

### Abbreviations

CHWs Community health workers.

NGOs Non-governmental Organizations.
