# Supplementary material for: Evaluating barriers to reaching women with public health information in remote communities in Mali
Source: BMC Health Serv Res. 2024 Aug 7;24:905. doi: 10.1186/s12913-024-11277-5 (PMC11308311; doi:10.1186/s12913-024-11277-5)
Supplement: Supplementary file 1 — Supplementary Material 1 [file 12913_2024_11277_MOESM1_ESM.pdf]

Supplementary Material 1

Interview & Focus Group Discussion Guide

A. One-to-one interview for women who have used the Viamo 3-2-1 service

Sources of health information

By health information, we mean guidance on such matters as what to do if your child has a fever, what the symptoms are of a disease, and what are the most effective methods of preventing illness.

1. What are the main sources of heath information in your area?
   Prompts: Radio, neighbours, mobile phone, community health workers, television, newspaper, community leaders, women's groups, health clinic.
2. Which of the mentioned sources of information do you trust the most? And why?
   Prompts: Radio, neighbours, mobile phone, community health workers, television, newspaper, community leaders, women's groups, health clinic, doctor, nurses, midwives

Improvements to services

3. Do you feel that you have enough information on malaria and how to get help if you/a family member falls sick? And why?
4. Do you feel that access to health information is an issue in your community? And why?
5. Do you ever feel that health information does not reach your community? If so, what improvements could be made?

Access to information

6. Which of the following do you own/have access to?
   - Radio
   - Television
   - Mobile phone
7. Which of these would be a preferred method for obtaining public health information, and would you feel that the information is current and trustworthy?
8. If you do not own a mobile phone, do you have access to one?
9. If free health information was provided through a mobile messaging service for free would you use it? And why?
10. Can you think of other ways that you think would be effective in providing health information?

Questions specific to Viamo 3-2-1 service

11. Did you find the health information provided by the Viamo 3-2-1 service useful?
12. Was the health information provided easy to understand?
13. What did you learn through the Viamo 3-2-1 service?
14. Would you use the Viamo 3-2-1 service again to get information about health and disease?
15. Please state why you would/would not use the platform again
16. Do you think that his mobile messaging service can be accessed by everyone in your community? If not why?

17. What recommendations can you provide to improve the service of the 3-2-1 mobile messaging platform?

B. One-to-one interview for women who have not used the Viamo 3-2-1 service

Sources of health information

By health information, we mean guidance on such matters as what to do if your child has a fever, what the symptoms are of a disease, and what are the most effective methods of preventing illness.

1. What are the main sources of heath information in your area?
   Prompts: Radio, Neighbours, Mobile phone, Community health workers, television, newspaper, community leaders, women's groups, health clinic).
2. Which of the mentioned sources of information do you trust the most? And why?
   Prompts: Radio, neighbours, mobile phone, community health workers, television, newspaper, community leaders, women's groups, health clinic, doctor, nurses, midwives

Improvements to services

3. Do you feel that you have enough information on malaria and how to get help if you/a family member falls sick? And why?
4. Do you feel that access to health information is an issue in your community? And why?
5. Do you ever feel that health information does not reach your community? If so, what improvements could be made?

Access to information

6. Which of the following do you own/have access to?
   - Radio
   - Television
   - Mobile phone
7. Which of these would be a preferred method for obtaining public health information, and would you feel that the information is current and trustworthy?
8. If you do not own a mobile phone, do you have access to one?
9. If free health information was provided through a mobile messaging service for free would you use it? And why?
10. Can you think of other ways that you think would be effective in providing health information?

Questions specific to Viamo 3-2-1 service

11. Did you know about the Viamo 3-2-1 mobile messaging campaign which was taking place in your community? YES/NO
12. If YES what was the reason that you did not use it?
    Prompts: No access to mobile phone, don't know how to use mobile phone, did not think it was useful, I thought I had to pay for it, I prefer to get health information from elsewhere, other reasons.
13. If NO, who would you like to have informed you of this initiative?
    Prompts: Community health worker, community leader, church, radio, others.
14. Do you think that this mobile messaging service can be accessed by everyone in your community? If not why?
15. What recommendations can you provide to improve the service of the 3-2-1 mobile messaging platform?

C. Focus group session with community leaders

Sources of health information

By health information, we mean guidance on such matters as what to do if a child has a fever, what the symptoms are of a disease, and what are the most effective methods of preventing illness.

1. What are the main sources of heath information in your area?
   Prompts: Radio, neighbours, mobile phone, community health workers, television, newspaper, community leaders, women's groups, health clinic.
2. Which of the mentioned sources of information do you trust the most? And why?
   Prompts: Radio, neighbours, mobile phone, community health workers, television, newspaper, community leaders, women's groups, health clinic, doctor, nurses, midwives.

Improvements to services

3. Do members of your community come to you if they need health information, and do you feel able to help them?
1. Do you feel that access to health information is an issue in your community? And why?
2. Do you ever feel that health information does not reach your community? If so, what improvements could be made?

Access to information

4. Which of the following do you feel most people in your community have access to?
   - Radio
   - Television
   - Mobile phone
5. Which of these do you think would be a preferred method for obtaining public health information, and would you feel that the information is current and trustworthy?
6. Do you own a mobile phone? If not do you have access to one?
7. If free health information was provided through a mobile messaging service for free would you use it? And why?
8. Do you think that other members of your community would benefit from this? And would it be something you are happy to recommend to your community?
9. How do you feel about the Viamo 3-2-1 mobile messaging service as a means to provide public health information to your community?
10. Do you think that the level of phone coverage is enough to reach everyone in the community? If not, who do you think would get left behind?
    Prompts: Men, women, young people, elderly, homeless persons, those with poor literacy, others.
11. Can you think of ways that Viamo can improve their service or other ways that you think would be effective in providing health information to women in your area?

D. Focus Group Discussion with Community Health Workers

Sources of health information

By health information, we mean guidance on such matters as what to do if a child has a fever, what the symptoms are of a disease, and what are the most effective methods of preventing illness.

1. What are the main sources of heath information in your area?
   Prompts: Radio, neighbours, mobile phone, community health workers, television, newspaper, community leaders, women's groups, health clinic.
2. Which of the mentioned sources of information do you trust the most? And why?
   Prompts: Radio, neighbours, mobile phone, community health workers, television, newspaper, community leaders, women's groups, health clinic, doctor, nurses, midwives.

Improvements to services

3. Do you feel that access to health information is an issue in your community? And why?
4. What do you think are the barriers to accessing health information in your community?
5. Have people in your community expressed a wish for more information, and if so how do you think they would like to receive it?

Access to information

6. Who are the hardest group of people to reach with health information? And why?
   Prompts: Men, women, young people, elderly, homeless persons, those with poor literacy, others.
7. What do you think are the main reasons for poor treatment seeking behaviour in your community? And why?
   Prompts: People don't have access to health information, people can't afford healthcare, people prefer to seek help from traditional healers, people don't want to take time off work
   people don't trust healthcare providers, health services are too far, other
8. Do you think that the Viamo 3-2-1 messaging service providing free health information is helpful for your community? And why?
9. Do you think that the level of phone coverage is enough to reach everyone in the community? If not, who do you think would get left behind?
   Prompts: Men, women, young people, elderly, homeless persons, those with poor literacy, others.
10. Could you think of ways that Viamo can improve the 3-2-1 messaging service to reach women in your area? Could you think of other ways that women in your area which may be more suited to reaching women with public health messages?
