# Supplementary material for: Evaluating barriers to reaching women with public health information in remote communities in Mali
Source: BMC Health Serv Res. 2024 Aug 7;24:905. doi: 10.1186/s12913-024-11277-5 (PMC11308311; doi:10.1186/s12913-024-11277-5)
Supplement: Supplementary file 2 — Supplementary Material 2 [file 12913_2024_11277_MOESM2_ESM.pdf]

Supplementary Material 2

Interview transcripts

Ouellessebougou, September 1, 2023

Group focus with the community health agents at the central community health center:

Bougouni-H-FG-1

Woman: What are the principal sources of health information in your community?
Number 1: Community members get health information from the radio.
Number 14: They get information from talk meetings hold at the community health center.
Number 9: Television is also their source of health information.
Woman: What do you trust most of all the sources you named ? Why?
Number 12: I trust the health information of the community health centers. It originates from doctors who are health professionals.
Number 1: I believe in the health information shared on the radio by doctors.
Number 15: It is radio. Radio shares information on time.
Number 2: I trust the radio, too.
Woman: is it a challenge for people to get health information in your community?
Number 13: People have access to health information, but some don't listen to the health information advice.
Number 1: It is a challenge. The community members attend talk meetings on health at the health center. The village relays go from families to families in remote villages to share health information with them. Despite this effort, some people dont follow the advice.
Number 14: Radios and local relays share health information with the community in remote areas.
Woman: Are all the expectations of your community on health issues met? Is there specific information on health that your community members need?
Obstetrician: At the maternity, we talk to pregnant women about health issues when they come for prenatal healthcare.
Number 9: During vaccination campaigns, we tell the community the importance of childhood vaccination. Also, during malnutrition activities, we discuss to help them learn more about it.
Number 1: The community members need to know more about preventive health care. They don't take the medicaments we give them for prevention.
Woman: Are they groups of people who can't access the health information in your community due to network or any other reason?
Number 9: Some farmers don't access health information. They are always busy working on farms. People in places with weak network coverage don't access health information, too. Some groups of people are reluctant to childhood vaccination despite the sensitization.
Number 14: Some women are still reluctant to attend health centers for baby delivery.
Woman: Why are many patients not attending health centers?
Number 15: They worry about how doctors welcome them.
Number 8: Some people can't afford the treatment at the health center.
Number 9: They don't have money to buy the medicaments.
Number 13: They refuse to go to the health center even if they can afford the expenses. They don't care about their health. They practice self-medication.

Woman: This project is a Viamo Service project. Some people call it Studio Tamani or Oumou Diarra's Talk Show. Viamo shares information on health, education, and cooking, marital affairs, to name a few. Have you ever learned about it?
Number 13: I have never heard about it.
Number 1: I heard about. Some people listen to it in my surroundings.
Number 3: I heard about it. I used to listen to it.
Woman: Did you find the Service information relevant?
Number 3: Yes, I did.
Woman: Are you still listening to it?
Number 3: No.
Woman: How have you learned about it?
Number 3: The service often calls to inform people about Oumou Diarra's Talk Show.
Woman: Five or six among you learned about the Service. Is there a network coverage issue in your community?
Number 9: There are network coverage issues in some places.
Woman: How many villages have network coverage issues?
Number 9: They are numerous.
Woman: Can people with no telephone borrow one to listen to the service in your community? Why?
Number 2: No, they can't. Phones are personal devices.
Number 3: Does the service work on both cellular and smartphones?
Woman: Yes, it does. I will give you the service number.
Number 3: Some people may accept to share their phone with someone. It depends on the person borrowing.
Woman: What to do to share health information with all your community members on time besides television, radio, and telephone?
Number 11: Health specialist should meet local organizations and discuss health issues with them in their community.
Number 13: I suggest sharing health information on popular radio talk shows.
Number 3: There is nothing to do. Much health information is on television, radio, and telephone, but some people are not interested.
Woman: We can't stop working. Much money is spent on health issues to help the community benefit from health. We want to see positive impacts on the ground.
Number 8: Even if you go from family to family to share health information, some will tell you they haven't received it.
Number 15: We need to do more.
Number 9: I am a vaccination agent. I go from village to village for vaccination campaigns. Before I go, local relays tell the community about my arrival, but some families tell me that they haven't got the information about it. Local relays should work hard to share health information with all the community members. They should go to mosques and everywhere to inform the community.
Woman: We are almost at the end of our conversation. Do you have any questions?
Numbers: We don't have any questions.

September 1, 2023

Conversation with the deputy of the Technical Director of the Health Center of Ouelessebougou:

Bougouni-H-I-1

Question: How old are you?
Answer: I am 40
Question: What is your sex?
Answer: I am a male
Question: What is your educational level?
Answer: I am a graduate State Nurse.
Question: What is your job?
Answer: I deal with medical advice.
Question: How many children do you have?
Answer: I have four children.
Question: I wish them a long life. Could you explain your duties and responsibilities at the community health center?
Answer: I am the deputy of the Technical Director of the Health Center. I deal with malnutrition issues.
Question: What are the principal sources of health information accessible to your community?
Answer: They are radios, television, and town criers.
Question: What sources do your community people trust much?
Answer: They trust the health information from the radio and town criers.
Question: Why does the community believe more in the radio and town criers?
Answer: They believe in the town criers because they know them. They are from the same community.
Question: Is it a challenge for people to get information about health in your community?
Answer: No, it isn't.
Question: Why do you think so?
Answer: There are 20 villages in our health district. In each, there are four local relay members. We share with them all the health information we get. In turn, they share it with their community people.
Question: What specific health topics should your community members learn more about on the service?
Answer: They should learn about childhood vaccination against malaria. Some don't like the vaccine to prevent malaria in children. They will say they haven't heard it when you share it on radio and television. Therefore, we ask town criers to go everywhere and inform all the community members.
Question: Are some specific groups of people not accessing health information in your community?
Answer: Yes. Young people and elders don't access health information.
Question: Why young people and elders don't access health information in your community?
Answer: They aren't targets of the health policy. We work with women a lot in the field of health.
Question: Why are many patients not attending health centers for treatment?
Answer: The reasons are the misunderstanding of community members of the importance of health, the way doctors welcome patients, and money issues.
Question: Viamo Service shares a lot of health information on the phone. You must have a minimum balance of CFA 100 to call the service, but it doesn't consume your balance in your account. Will they listen to the service if they don't have to pay a minimum balance?
Answer: Yes, they will. They won't accept to pay even a minimum unconsumed balance of CFA 25. They may think it is lucrative.
Question: Is network coverage an obstacle to calling the Viamo Service?

Answer: I don't think network coverage will be an obstacle. There is network coverage almost everywhere today. In the area of Ouelessebougou, about 44 villages, there is network coverage.

Question: What to do to convince women to call the service?

Answer: You can inform them about the health content on Studio Tamani during "Baroni" time. Many women listen to "Baroni". They are very interested in it. It is amusing for them. You can also broadcast the Service health information before "Baroni" starts.

Question: What to do to share health information with women?

Answer: You can choose some people who will talk about health information for about 15 minutes before prenatal and postnatal healthcare when many women attend the health centers. You can also register the health information on flashcards or drivers that women can watch on TV during their health meetings at the health center.

Question: We are almost at the end of our conversation. Do you have any suggestions?

Answer: I suggest you register the Viamo Service health content on flashcards or drivers and give it to doctors. Doctors can help the community members access them during periodic health meetings on TV at the health center. Thank you very much for what you are doing.

Question: You are welcome. See you!

Answer: See you!

September 1, 2023

Conversation with the midwife and the deputy of the Technical Director of the Central Community Health Center of Ouelessebougou: Bougouni-H-I-2

Question: How old are you?
Answer: I am 36
Question: What is your sex?
Answer: I am a male.
Question: Did you attend school?
Answer: Yes, I did.
Question: What grade did you reach?
Answer: I am a university graduate.
Question: What is your occupation?
Answer: I am a midwife and the deputy of the Technical Director of the Center.
Question: How many children do you have?
Answer: No, I don't have any.
Question: I wish you good luck. What is your role in the community?
Answer: I help women in baby delivery, prenatal healthcare, and family planning and write monthly health reports.
Question: How do people get health information in your community?
Answer: They get it from local relays, radio, television, social media, and female organizations.
Question: How do they get health information from female organizations?
Answer: During the meetings of female organizations, they talk about sharing some health information.
Question: Of the sources you cited, which do community members trust most?
Answer: They trust the local relays, the community health agents, television, and radio.
Question: Why do they trust them most?
Answer: They trust them because they come from a good source.
Question: Is it a challenge for people to get health information here?
Answer: Yes, it is. Sometimes, leaders of female organizations can't share health information with members. Members are in a hurry to go back to their activities. They don't have time to listen to radio and television. They are all attracted by phones. Sometimes, they listen to "Baroni."
Question: Are people in your community expressing their needs for specific health information?
Answer: It will be interesting for them to get more information on the childhood vaccination against malaria. They are reluctant because of the side effects of the vaccine. Many community members understand the importance of childhood vaccination, except the malaria vaccine.
Question: Are there some groups of people who don't have access to health information?
Answer: Yes, there are. Men don't have access to health information. They don't have time to listen to it. They are always working.
Question: What activities do people perform here?
Answer: They practice farming and trading.
Question: What are the reasons why some people don't attend health centers for treatment on time?
Answer: Distance is a challenge in the rainy seasons. Lack of money is a reason, too.
Question: Have you ever heard about health information on Studio Tamani?
Answer: No, I haven't.
Question: Viamo Service shares a lot of health information on the phone. You must have a minimum balance of CFA 100 to call the service, but it doesn't consume your balance in your account. Will they listen to the service if they don't have to pay a minimum balance?

Answer: Yes, they will. But I suggest informing people about the service on TV and the radio. Many people don’t know about the Service. As health workers, we should be the first to know about it, but many don’t.

Question: Is network coverage available in your community to call the Viamo Service?

Answer: There is no network coverage everywhere. There is a village located eight kilometers from here with no network coverage.

There is network coverage almost everywhere today. In the area of Ouelessebougou, about 44 villages, there is network coverage. There are some specific places where there is a network. Community members can go there to call the Service if they learn about it.

Question: What do you suggest to share health information with all the community members via the Service?

Answer: I suggest sharing the content of its health information on radio partners during “Baroni.” Women like to listen to “Baroni” a lot.

Question: What to do to convince women to call the service?

Answer: Inform them about the health content on Studio Tamani first. Make the Service calling free of charge.

Question: We are almost at the end of our conversation. Do you have any suggestions?

Answer: I suggest discussing the childhood vaccination against malaria when broadcasting “Baroni.” People in Ouelessebougou don’t understand the advantages of childhood vaccination against malaria. They refuse kids to take the vaccine.

Question: Thank you very much for your attention.

Answer: You are welcome!

September 1, 2023

Conversation with the Community Health Agent in Ferekoroba.

Bougouni-H-I-3

Question: Hello Madam. How are you?
Answer: I am fine, thanks.
Question: How do you get health information?
Answer: We mostly get it from radio, television, phones, and social media.
Question: What is your name?
Answer: I am Awa TRAORE
Question: How old are you?
Answer: I am 31
Question: Did you attend school?
Answer: Yes, I did.
Question: What educational level did you reach?
Answer: Secondary school level.
Question: What is your occupation?
Answer: I am a Community Health Agent.
Question: How many children do you have?
Answer: I have got three children.
Question: How do you get health information on malaria?
Answer: We get it from radio, television, and Studio Tamani. Community Health Agents also share it during talk meetings.
Question: What are the most reliable or credible sources of information your community trusts?
Answer: If only health agents share health information with them, they may not trust it. But, when they hear the same information on Studio Tamani, they will believe in it.
Question: Do they face challenges in getting information on health through the means you cited?
Answer: It may be difficult due to how to manipulate the device.
Question: What to do to face that difficulty?
Answer: You can sensitize them or show them how it works.
Question: Do all your community members have access to health information on time?
Answer: No. Some don't listen to the radio and television. Others don't know about the service.
Question: Do community members want to have access to health information differently?
Answer: Yes, they like talk meetings on health a lot.
Question: Do they express their need to get a piece of specific health information?
Answer: Yes, they do it from time to time.
Question: Do you remember an example when someone goes to see you to seek health information?
Answer: Yes. Someone used to ask me about mosquitoes. The woman wanted to know how to distinguish a male mosquito from a female mosquito that transmits malaria.
Question: Are some groups of people who don't get health information?
Answer: Yes. Young girls and elders don't have access to health information. Some elders don't let young people listen to some health issues.
Question: What difficulties do you still have attending health centers to heal malaria?
Answer: Sharing accurate information on health with the community members: sleeping under mosquito nets
Question: Why are many patients not attending health centers to heal malaria?
Answer: Lack of money is the principal reason.

Question: Are talk meetings on health important? If yes, why?
Answer: People attending them learn more.
Question: What did you benefit from the talk show on the phone?
Answer: I have learned more about family planning: what to do if you forget to take your pills.
Question: Can many women use phones to get health information in your community?
Answer: No, they can't because of the weak network coverage.
Question: Who are the most impacted by the weak network coverage?
Answer: Young people and elders. We are working with phones a lot. The lack of a network is a hardship for us.
Question: What do you suggest the service to do for improvement?
Answer: You can share health information with young people and elders on tapes during talk meetings. You can also give them a radio or flashcards containing the registered information.
Question: What to do to encourage husbands and wives to listen to the service together on the phone?
Answer: You can sensitize men and women on the importance of the service.
Question: What specific health issues should your community members learn more about?
Answer: They should learn about the disadvantages of early marriage.
Question: What is the early age of girls' marriage in your community?
Answer: 14, 15 years old.
Question: What do you suggest to make the Viamo Service popular?
Answer: You can inform people about it and tell them how it works during the talk shows
Question: What to do to make groups of people listen to the service together and get benefits?
Answer: You should talk about the importance of the service to them.
Question: What to do for the follow-up of Viamo Service after the end of the project?
Answer: I suggest selecting some leaders in each village. They will be in charge of the follow-up. But, they need to be trained on health issues first.
Question: Do you have any suggestions?
Answer: I suggest you meet elders and young people and sensitize them to listen together to the service. I hope that they listen to you. We are familiar with them. They may not listen to us, but they would listen to you more.
Question: Do you have any questions?
Answer: No, I don't.
Question: OK, this is the end of our conversation. Thank you very much for your attention.
Answer: You are welcome.

Beneko, September 6, 2023

Conversation with a Community Health Agent in the health district center of Ouelessebougou:

Bougouni-H-I-4

Question: Good morning, Sir. How are you?
Answer: I am fine, thanks.
Question: We were supposed to meet you in Beneko at your health center. But because of the bad condition of the road we talked on the phone. Our conversation will focus on health information. Orange Mobile Operator and Studio Tamani are collaborating to share health information with community members to empower people with health information. Have you ever heard about it?
Answer: Yes.
Question: How old are you?
Answer: I am 33
Question: Did you attend school?
Answer: Yes, I did.
Question: What educational level did you reach?
Answer: Secondary school level.
Question: What is your occupation?
Answer: I am a Community Health Agent.
Question: Do you practice any remunerative activity?
Answer: Yes, I do. I grow farms.
Question: How many children do you have?
Answer: I have three children.
Question: I wish them a long life. What is your role in the community?
Answer: Thanks. I am sensitizing the community members to help them with disease prevention. I talk about family planning with women. I also told them to go to the health center early in case of malaria.
Question: We need your consent for this conversation. Do you agree to talk with us about health?
Answer: Yes, I do.
Question: What are the sources of health information accessing your community members?
Answer: They get it from radio and television. Community Health Agents also share it during talk meetings.
Question: What are the most reliable or credible sources of health information in your community?
Answer: They trust the community health agents share with them.
Question: Why do they trust community health agents?
Answer: They trust us because we are familiar with them. They get in touch with us for any health issues.
Question: Is it a challenge to get health information in your community?
Answer: Sometimes, the health agents get health information late, and the community can’t get it.
Question: Why do you get health information late sometimes?
Answer: Health agents get most of their health information from the Center's Technical Director and during workshops. We can spend a long time without any workshops.
Question: What other challenges do you have in getting health information?
Answer: Some elders don’t get health information because they don’t listen to radio and television.
Question: Do your community members express their needs to get health information? If yes, how do they want to access health information?

Answer: Yes. During our talk meetings on health, some expressed their need to learn about a particular health issue.
Question: Could you give an example of a particular health topic they are interested in?
Answer: Women are more interested in family planning. They want to attend talk meetings together with their husbands because some men are reluctant on the topic. They express their need to learn more about malaria in the rainy season.
Question: What are the most difficult groups of people to share health information in your community?
Answer: No groups of people who don't access health information. They all access it.
Question: How do you share health information with your community members?
Answer: I go from family to family to talk with them about health information. I also attend the weekly meetings of female organizations to share on health issues.
Question: Do you target some specific group of people when sharing health information?
Answer: We don't talk about some sensitive topics with elders only. But with others, we can talk about all health issues.
Question: Why are many patients not attending health centers for treatment?
Answer: Lack of money is the principal reason. They all say they go to the health center late because of money issues to buy the medicaments. It is up to the family chief to order to send one of his family members to the health center for treatment. When he can't afford it, they go to traditional healers.
Question: Are women practicing remunerative jobs to participate in the expenses of the family health issue?
Answer: Most of them work on farms. They are practicing remunerative jobs, but they can't afford health expenses.
Question: Will women send their kids to health centers if they can afford the health expenses?
Answer: Yes, they will.
when they have money,
Question: What do you know about Studio Tamani?
Answer: I can't say much about it, but my wife can. I called the service for her to listen to Oumou Diarra's Talk Show.
Question: Can you tell me how to access Studio Tamani?
Answer: You dial 37321 to listen to Studio Tamani. The service itself guides you to select what you want to listen to. You press 1 for health information, for newsreel, you press 3, and so forth.
Question: Why don't you listen to the service yourself?
Answer: It is beneficial, but I don't have enough time to listen to the service.
Question: How did you learn about the service?
Answer: I learned about it via a message sent by an Orange phone operator.
Question: Have many people learned about Oumou Diarra's Talk Show in your community?
Answer: No, they haven't. Some people listen to Studio Tamani's newsreel only.
Question: Why people didn't learn about it despite the effort of the Orange operator to inform them via messages?
Answer: Illiteracy is one of the reasons. Most people can't read messages.
Question: What to do to inform them about the service?
Answer: Community health agents and local relays can inform people about the Viamo Service.
Question: Who has more phones in your community, men or women?
Answer: Most people have phones.
Question: What to do to inform the few people without phones about the Service?
Answer: They can listen together on the same phone or watch television.
Question: What group of people don't have phones?

Answer: Females, because they can't keep a phone.
Question: What to do to encourage husbands and wives to listen to the service together on the phone?
Answer: You can sensitize men and women to listen to the service together.
Question: What to do to convince them to listen to the service together on the phone?
Answer: You must sensitize them.
Question: Who can sensitize them to listen to the service together?
Answer: Local relays and community health agents can do the job in my area.
Question: What is the name of your area?
Answer: It is Zelabougou.
Question: How long is it from Beneko?
Answer: It is six km.
Question: What specific health topics should your community members learn more about on the service?
Answer: They should learn about malaria. Malaria is a local issue here. In the rainy seasons, many people catch it.
Question: What else do you suggest as specific health topics?
Answer: I suggest prenatal and postnatal healthcare, childhood vaccination and family planning, early marriage, and excision.
Question: Are people still practicing excision in your area?
Answer: Yes, they are. But the number of excisions is decreasing now.
Question: What is the early age of girls' marriage in your area?
Answer: The earliest age is 14 or 15.
Question: What to do to convince women and family members to get interested in the service?
Answer: It depends on information and sensitization. Women will get interested in it if we tell them the importance of the platform.
Question: What to do to make groups of people listen to the service together to benefit from the health information?
Answer: In the rainy season, people here are busy on the farms all day. They can listen to the service around 6 p.m. only.
Question: What to do to make groups of people listen to the service together and benefit?
Answer: You can call for meetings to make them listen to the service together.
Question: What to do for the follow-up of Viamo Service after the end of the project?
Answer: I suggest to reward the best listeners of the service. It will encourage others to call the service.
Question: Do you have any suggestions to improve Service 37321?
Answer: I suggest sharing the same information available on Service 37321 on the radio. People who don't have phones will use their radio to access it. Many women listen to "Baroni" on the radio here.
Question: What other means of communication do you suggest to share health information with women besides radio and telephone?
Answer: I suggest local relays and community health agents.
Question: We are almost at the end of our conversation. Do you have any suggestions?
Answer: As community health agents, we work for the community's well-being. We need to get health information on time. The community members will learn about it as soon as we get the information.
Question: Thank you very much for your attention.
Answer: You are welcome.

Ferekoroba, September 2, 2023

Group focus with the community authorities:

Bougouni-L-FG-1

Man: Question 1: Question 1: How do you get health information in your community?
Number 8: We get our health information from the community health center, radio, and women who attend the health centers for vaccination, malnutrition, and prenatal healthcare. Last night, I listened to a show about kids and pregnant women's nutrition on the radio.
Number 3: We get our health information from the local radio. Those who attend health centers share with us what they learn there. The dentist of our health center used to talk about dental caries. I learned a lot from him about dental caries.
Man: A part from radios where do you get health information?
Number 4: Radio listeners share health information with others, too.
Number 5: As they all said, radio is our most reliable source of health information. Local relays are also a source of information.
Man: Which source do you trust most? Why do you trust it?
Number 4: We trust doctors and local relays. I trust them because I go to the health center for treatment. They are health professionals.
Number 3: I trust local relays and doctors. During the malaria prevention campaign, four of my children took some tablets for malaria prevention, and now they have caught malaria.
Number 1: I trust doctors because, before the arrival of Abba in our community health center, we stopped sending our patients to the surrounding health centers. He talks to women on health issues.
Number 6: We trust Doctor Abba. We brought health to our community.
Man: Do you think you have more health information to share with people in need in your community?
Number 5: We share health information with people in need, but we don't know if they are satisfied.
Number 8: We are not healers but share what we learned from our ancestors.
Anonymous: As he said, young people reach elders to learn about health issues. Some go to the traditional healers.
Man: Are you facing challenges to get health information in your area?
Number 8: We are not facing any challenges to get health information because of local relays and radios.
Number 5: I agree with Number 8.
Man: Does it happen to you to wonder whether your community members access health information regarding their behavior? If yes, what do you advise?
Number 5: We suggest periodic talk meetings on health issues with women. Talk meetings will help women learn more about health issues.
Man: I don't think you got my question. I said, despite sharing the health information, are you satisfied with your community members in the health field regarding their behavior? What do you suggest to do?
Number 6: We trust all the health information. When we follow the advice of doctors, we protect ourselves against diseases.
Number 5: Villagers are different from citizens. Villagers listen to health information but don't care about it as citizens.
Number 6: Radios and televisions sensitize the community on childhood healthcare. We learned from them that children must not play in dirty puddles and must sleep under mosquito nets to avoid malaria.

Man: Which source of information is easy to access in your community among radio, television, and telephone?

Number 8: It is radio. I listen to the health information on “Radio Renouveau” broadcasted by our local radio “Faso Kanou”.

Number 3: It is radio. Many people don’t watch television or use the telephone.

Number 5: It’s radio because people don’t know if we can get health information on phones.

Man: What do you trust more, television, radio, and telephone? Why?

Number 7: We trust television because its source of information is doctors.

Anonymous: I trust television.

Number 1: I listen to the radio. I believe in it because their advice is efficient. I don’t watch television.

Number 8: We believe in radio because their information comes from doctors. We believe in our local doctors.

Man: Do you all attending this meeting have phones?

Number 1,2,3,4,5,6,7,8: We all have phones.

Man: Studio Tamani broadcasts much health information. You can call the service to get relevant health information. Will many people call it if it is free?

Number 8: I call the service to listen to health information regularly.

Numbers: We all get phones but don’t call the service to listen to them.

Woman: You can call the service for free, on condition that you have a balance of CFA 100 in your phone account Orange chip. Will more people call the service when they don’t have to pay a minimum balance of CFA 100 in their phone account?

Number 8: I used to call the service but haven’t noticed if it consumes balance.

Anonymous: You should teach people how to call the service. They don’t know how it works. They will use it if they know how it works because more people have telephones.

Number 3: I don’t listen to Studio Tamani because many sources of information are available today. You can’t listen to all of them.

Man: What do you think about this initiative to broadcast health information on phones?

Number 5: Sharing health information on phones is more accessible to people than other ways. You need to inform people how to listen to health information on phones. You can call the service anytime for health information with phones. But, on televisions and radios, there is a time set for health information. You can miss it sometime.

Number 8: It is easy to access information on phones. When you call, it guides you to select your need by pressing numbers. I call it to listen to the newsreel and health information. Also, you can call the service a cellular or a smartphone.

Man: Do all your community members, men, women, elders, youth, and illiterates have access to network coverage?

Number 4: We face network issues during rainy seasons in some places. There are places where it is accessible.

Number 8: Network accessibility depends on places. In some areas, it is good, but in others it is not.

Man: What do you suggest to improve the service on the phone?

Number 4: The service on the phone depends on network coverage. The network must be available first.

Number 3: We need a good network to improve the service.

Question from woman: Females need more health information on kids' healthcare and family health care, but only a few have phones. What to do, especially for women, to listen to the service with their husbands?

Number 8: Many women have phones here but don’t call the service to listen to health information. Also, they have radios but are not interested in health information. They need sensitization to the importance of health information.

Number 4: Women are not interested in health information shared by phones and radios.

Number 3: Women have phones but don’t always use them. You can call ten women, but only three out of ten will probably pick up the phone.

Woman: What to do to get women interested in the health information on the service?

Number 3: There is a high illiteracy rate here. I receive many messages myself, but I can’t read them. Women need to learn how to read and write to use the service.

Number 5: The service on the phone is something new. You need to inform people about it.

Woman: Which health topic do you want to learn more about?

Number 5: We want to learn more about malaria. Many people catch it in the rainy season.

Number 7: Husbands can sensitize their wives to listen to health information. The majority of women having phones here don't listen to health information.

Number 4: We need a good wife to care for our family. Men spend all day at the workplace. They don’t have time to take care of the kid’s health. When a kid catches malaria, you can waste CFA 25,000 to heal him at the health center.

Man: It is almost the end of our conversation. Do you have any questions?

Anonymous: I want to know how Number 8 learned about the service.

Number 8: I learned about the service by chance while manipulating my phone four years ago. They shared a number. I registered it and called it.

Man: Why did you not inform others?

Number 8: I did inform people. I listen to it with others.

Man: Listen to it together with your wife next time.

Ferekoroba, September 2, 2023

Group focus on women who are not using Viamo Service

Bougouni-NU-FG-1

Question: How do people get health information in your community?
Number 6: We get health information from doctors, radio, and telephones.
Number 7: We get health information from the radio.
Number 4: We get it from some talk show on the radio.
Question: What sources of information do you trust among radio, telephone, and television?
Number 5: I believe in radio
Number 7: I trust the radio because it shares relevant information.
Number 3: I trust the radio.
Question: What do you know about malaria?
Number 7: Mosquito transmits malaria. Dirty places attract mosquitoes.
Number 8: Some foods can transmit malaria.
Question: Do you think you know more about malaria?
Number 6: We can avoid getting malaria when listening to the advice.
Number 3: We need to listen to the sensitization on malaria prevention: sleeping under mosquito nets and cleaning dirty places.
Question: Is it a challenge for people to get health information in your area?
Number 6: It is not a challenge. Doctors are available in case of health information. Radio and telephone share health information, too.
Number 7: It is not a challenge. We need to go to Ouelessebougou for health issues. It is a challenge for us.
Number 5: It is not a challenge. Doctors share health information with community members.
Number 3: I agree with number 5.
Number 8: It is not a challenge, but some Mothers stop giving medications to kids after a few days of treatment. It is not a good attitude.
Question: Are there some groups of people who don't have access to health information due to disability, age, sex, or any other reason?
Number 8: No group of people is lacking health information in our community.
Question: What is your most used device to get health information from radio, telephone, and television?
Number 2: It is radio.
Number 7: It is radio, too.
Number 2: I said radio because it is accessible everywhere and anytime. We watch TV only at night.
Number 3: It is radio because it is accessible.
Question: Do all of you attending this conversation have phones?
Number 3: Yes, I do.
Number 2: Yes. I have a phone.
Number 8: I have a phone, too.
Number 7: My phone doesn’t work.
Number 6: I have a phone.
Number 1: I have a phone.
Question: Two out of eight don’t have a phone. Is it possible for you to borrow a phone to listen to health information?
Number 7: No, it is not.

Number 5: I agree with her.
Number 3: People don't use the telephone to listen to information in our community. They all listen to the radio.
Number 8: It is not possible to borrow it.
Question: Despite the availability of health information on radio, television, and telephone, some people still don't access it. What to do to share health information with all community members?
Number 3: I suggest talk meetings to share health information. Town criers can inform people about the meeting.
Number 8: Even if you call a meeting to discuss health issues, some people will not attend it. They would prefer to go shopping.
Question: What to do to motivate them to attend the meeting?
Number 8: I suggest finding those who won't attend meetings about health issues.
Question: The Viamo Service contains a lot of health information**.** It is available on telephones. It is free to call the service with an orange chip even though you need to have a minimum balance of CFA 100. Despite the large amount of health information on the Viamo Service, community members didn't learn it. What do you suggest to share health information on, especially for women?
Number 7: I suggest the doctors of the health center.
Number 3: I suggest doctors, too.
Question: What do you suggest to do to reach all the community with health information?
Question: Husbands have telephones more than wives**.** What to do to encourage husbands and wives to listen to the service together on the phone?
Number 2: It is not possible. There is no solution to that. They don't accept it.
Number 3: Men and women aren't constantly together in the same place. In rainy seasons, men work on farms while women work on their groundnut or rice farms. They are not in the same place.
Number 7: The couple can't listen to the health information together on the phone.
Question: What specific health topic do you want to learn more about?
Number 3: We need more information on malaria
Number 6: I suggest more talk shows on health.
Number 7: I suggest talking about how to use mosquito nets.
Number 8: I suggest telling people to complete a treatment. Some stop taking medication a few days later when they start feeling well.
Question: What to do to make people call the service on the phone to listen to health information?
Number 3: Some people don't listen to the service because they don't have a phone.
Question: About ten women can gather and listen to the service together. What do you think to encourage people to listen to health information together?
Number 6: It is difficult for women to gather and listen to the same phone. I suggest talking about meetings on health with health professionals instead.
Number 7: Women can listen to the service when they gather for group work activities on farms.
Number 8: Doctors can call for meetings about health issues with the community members.
Question: What do you suggest to do to reach all the community with health information?
Number 2: I suggest sharing the health information on the radio
Number 3: I advise sharing health information on phones. They are efficient tools for sharing health information. You can move with them and use them everywhere.
Question: We are at the end of our conversation. Do you have any questions?
Number 3: Thank you for what you are doing for the communities. We like talk meetings a lot because we learn from them.
Number 7: Thank you for this conversation on health. We learn more with the talks on health.
Question: Thank you very much for your attention. See you next time.

September 2, 2023

Conversation with a woman who is not using Viamo Service in Ferekoroba, Health District of Ouelessebougou:

Bougouni-NU-I-1

Question: How old are you?
Answer: I am 28
Question: Did you attend school?
Answer: No, I didn’t.
Question: What is your occupation?
Answer: I am a farmer.
Question: Do you practice any remunerative activity?
Answer: Yes, I do. I weave to get some money.
Question: How many children do you have?
Answer: I have three children.
Question: I wish them a long life. How do you get health information in your village?
Answer: Thanks. We get it from the doctors and the literacy classes. I am attending adult literacy classes now. We also talk about health issues there.
Question: How do you get health information about childhood vaccination campaigns?
Answer: Doctors go from family to family to inform the community members.
Question: What are the most reliable or credible sources of health information according to you?
Answer: I trust all of them because I learn a lot from them.
Question: What do you know about malaria?
Answer: When you catch malaria, you feel like drinking too much water. You feel lazy. It changes the color of some children’s eyes.
Question: Do you think you know more about malaria?
Answer: Yes, I do.
Question: Why do you think you know more about malaria?
Answer: Because we are taught much information about it.
Question: Do people in your community have difficulties getting health information?
Answer: Yes. Some don’t listen to the advice on health issues.
Question: Do you think that all the members of your community get health information?
Answer: No, those who don’t attend adult literacy classes don’t.
Question: Aren’t people here who inform families during childhood vaccination campaigns?
Answer: Yes. Some people go from family to family to inform them. But, it is not regular.
Question: Are there groups of people who don’t have access to health information in your community?
Answer: Yes, there are. Young girls don’t have access to health information.
Question: Why don’t they have access to health information?
Answer: Because people don’t give the health information. Ignorance is also one reason.
Question: What means of communication are the most used by people to get health information in your community?
Answer: Radio is the most listened to.
Question: What about telephone and television?
Answer: Some people have them, but radio is the most used.
Question: What means of communication would people prefer to get health information in your community?

Answer: It depends on the financial means of people. They would prefer radio and television because they give more explanation.
Question: Do you have a telephone?
Answer: Yes, I do.
Question: Do many women have telephones in your community?
Answer: Some have, but others haven't.
Question: Is the number of women with phones higher or not?
Answer: Most of the women here have phones.
Question: Can people with no phones borrow from others to listen to health information?
Answer: It is possible, provided the owner is not working with her phone.
Question: It is free to call the service even though you need to have a minimum balance of CFA 100. It doesn't consume your balance in your account. Will more people call the service if they don't have to get a CFA 100 balance in their account?
Answer: Yes, they will.
Question: Why do you think that more people will call it?
Answer: Sometimes, you don't have any balance in your account. It means you can't call it even though you want to listen to your information.
Question: What other ways do you suggest to reach all the community members with health information besides telephone, radio, television, and doctors?
Answer: I suggest some talk meetings on health issues. Those attending them will learn about health issues.
Question: Have you ever heard about the Viamo Service?
Answer: No, I haven't.
Question: Who do you think should give you health information?
Answer: Our midwife at the health center. Sometimes, she attends our adult literacy classes and shares some health information with the learners.
Question: Why do you say the health information is not accessible to all the community members?
Answer: You can't listen to it because you don't have a minimum balance of CFA 100.
Question: What to do to make people listen to the health information on the service?
Answer: You should plan to talk meetings and inform women about the service.
Question: What to do to encourage husbands and wives to listen to the service together on the phone?
Answer: Men here won't accept to lend their phones to their wives, but you can try to sensitize them.
Question: Who can sensitize men to accept it?
Answer: Maybe elders.
Question: What special health issues do your community members want to learn more about?
Answer: We want to learn more about malaria. Malaria is a local issue here. We want to learn how to avoid getting it and what to do in case of malaria.
Question: What to do to make women listen to the service and get benefits?
Answer: You need to spread the information on radio and television to inform them about the service.
Question: What to do for women to listen to the service together on the phone?
Answer: We should find someone to gather women to listen together according to a schedule.
Question: Who can deal with the follow-up of the service activity in your community?
Answer: You should look for the right person to do it. Some can start but not continue.
Question: Do you have any questions?
Answer: No, I don't. I just want to tell you that we need people to come and talk with us about health from time to time.
Question: OK, this is the end of our conversation. Thank you very much for coming to talk with us.

Answer: You are welcome.

Beneko, September 2, 2023

Conversation with a woman who is not using Viamo Service; Health District of Ouelessebougou:

Bougouni-NU-I-2

Question: How old are you?
Answer: I am 35
Question: Did you attend school?
Answer: No, I didn’t.
Question: What is your occupation?
Answer: I am a housekeeper.
Question: Do you practice any remunerative activity?
Answer: Yes, I do. I sell some donuts to get some money
Question: How many children do you have?
Answer: I have ten children.
Question: Are they all alive?
Answer: No, nine are alive.
Question: How do you get health information in your village?
Answer: We mostly get it from the health center.
Question: How else do you get health information in your village?
Answer: I don’t remember any.
Question: Do you find reliable or credible health information shared by doctors?
Answer: I find them credible information. You get healthy when you listen to them.
Question: Do your community members get enough health information?
Answer: Yes, they do.
Question: Why do you think that?
Answer: Because people practice what they know about health information.
Question: Do you have any difficulties getting health information?
Answer: No, we don’t.
Question: Who gives you health information?
Answer: Molobaly, Barou, and Hama are our doctors. They give us health information any time we are in need.
Question: Do all your community members have access to health information on time?
Answer: Yes, they do.
Question: What about those in the surrounding places?
Answer: They get health information, too.
Question: What means of communication do people use to get health information in your community?
Answer: They get health information from radio and television. I listen to the radio to get health information.
Question: What means of communication are the most used by people to get health information in your community?
Answer: Radio is the most listened to. Many women listen to” Baroni” here.
Question: Do you have a telephone?
Answer: Yes, I do.
Question: Can people with no phones borrow from others to listen to health information?
Answer: It is not possible, because everybody needs his phone.

Question: It is free to call the service even though you need to have a minimum balance of CFA 100. It doesn't consume your balance in your account. Will more people call the service if they don't have to get a CFA 100 balance in their account?
Answer: Yes, they will.
Question: Some people are complaining about not getting information during childhood vaccination. What to do to inform all the community members about health information?
Answer: Radio shares information on health, but women are busy working on their farms.
Question: Have you ever heard about the Viamo Service?
Answer: No, I haven't.
Question: Would you like to learn how to work?
Answer: Yes, I would.
Question: Who do you think should give you health information?
Answer: Our midwife at the health center.
Question: What to do to make people listen to the health information on the service?
Answer: You should tell them how it works.
Question: What other ways do you suggest to reach all the women and youth of the community with health information?
Answer: Women have radio and telephone. But, they don't get health information because of farming activities.
Question: What to do to encourage husbands and wives to listen to the service together on the phone?
Answer: Men here won't accept it, but you try to sensitize them.
Question: What special health issues do your community members want to learn more about?
Answer: They want to learn more about malaria.
Question: What to do to make more people call the service for health information?
Answer: I suggest to inform them about the service.
Question: What can make women listen to the service together on the phone?
Answer: You need to call for talk meetings to discuss with women.
Question: Do you have any questions?
Answer: No, I don't.
Question: OK, this is the end of our conversation. Thank you very much for your attention.
Answer: You are welcome.

Beneko, September 2, 2023
Conversation with a woman who is not using the Viamo Service: Bougouni-NU-I-3

Question: How old are you?
Answer: I am 30
Question: Did you attend school?
Answer: Yes, I did.
Question: What educational level did you reach?
Answer: Grade seven.
Question: What is your occupation?
Answer: I am a housekeeper.
Question: Do you practice any remunerative activity?
Answer: No, I don’t.
Question: How many children do you have?
Answer: I have four children.
Question: Are they all alive?
Answer: Yes, they are.
Question: How do you get health information?
Answer: We mostly get it from radio and television.
Question: What are the most reliable or credible sources of information according to you?
Answer: Doctors, radio, and television are the most reliable or credible sources.
Question: Do you get enough health information?
Answer: Yes, we do.
Question: Do you face challenges in getting health information?
Answer: No, we don’t.
Question: Do all your community members have access to health information on time?
Answer: No. Some don’t get health information.
Question: What to do to help all the community members get health information?
Answer: You can sensitize them to attend health centers and listen to radio and television.
Question: Are some groups of people who don’t get health information?
Answer: Yes. Young people don’t have access to health information.
Question: What to do to help young people get health information?
Answer: You can teach them about health issues.
Question: What means of communication do you use to get health information?
Answer: I listen to the radio.
Question: Does radio share accurate health information?
Answer: Yes, it does.
Question: Do you have a telephone?
Answer: Yes, I do.
Question: Why don’t you use your telephone to get health information?
Answer: It is easier with the radio.
Question: There is a lot of information on the telephone for free: Oumou Diarra’s Talk Show, entertainment, health, etc. Will you call the service if you don’t have to pay for an unconsumed CFA 100 balance to listen to health information?
Answer: Yes, I will.
Question: Why will you listen to it?

Answer: Because you learn something when you listen to it.
Question: What other ways do you suggest to reach all the community members with health information?
Answer: I don’t have any idea.
Question: You dial 37321 to listen to the health information. You must have a minimum unconsumed balance of CFA 100 during your call. The service is free. Why haven't you called it yet?
Answer: I didn’t know about it.
Question: Do you think that calling the service is easy for everybody?
Answer: Yes, it is. There is good network coverage now.
Question: What to do to improve this job?
Answer: You should keep on working hard.
Question: What to do to encourage husbands and wives to listen to the service together on the phone?
Answer: You need to sensitize men.
Question: Among the following: Oumou Diarra’s Talk Show, entertainment, health, etc. What do you advise husbands and wives to listen together?
Answer: I suggest they listen to health information.
Question: What to do to inform women and their families about the Viamo Service?
Answer: I suggest to inform them on radio and television.
Question: What can be the impact of the Viamo Service information on health preservation?
Answer: listeners will practice more sanitation measures.

September 2, 2023

Conversation with a woman who is not using Viamo Service in Beneko, Health District of Ouelessebougou:

Bougouni-NU-I-4

Question: How old are you?
Answer: I am 36
Question: Did you attend school?
Answer: No, I didn't.
Question: What is your occupation?
Answer: I am a farmer.
Question: Do you practice any remunerative activity?
Answer: Yes, I do. I make shea butter.
Question: How many children do you have?
Answer: I have six children.
Question: I wish them a long life. **:**
Answer: Thanks.
Question: Our conversation will focus on health information. How do people get it, and how do they catch malaria? How to avoid getting malaria, what to do in case of malaria, what to do when your kid is vomiting or has high temperature?
Answer: OK.
Question: How do you get health information in your village?
Answer: We get it from the doctors, television, and radio.
Question: What are the most reliable or credible sources of health information according to you? Why?
Answer: I trust doctors. You feel healthy when you listen to doctors. I believe in television, too.
Question: Do you think you know more about malaria?
Answer: Yes, I do.
Question: What do you know about malaria?
Answer: Mosquitoes transmit malaria. Dirty water puddles are also causing it.
Question: Do people in your community have difficulties getting health information?
Answer: No, they don't.
Question: Why do you think people in your community have no difficulties getting health information?
Answer: Doctors are accessible. They share health information during prenatal healthcare and childhood vaccination campaigns.
Question: Do you think that all the members of your community get health information?
Answer: Maybe people in villages without health centers don't get it. Radio and television can help them get it.
Question: What means of communication do you have?
Answer: I have radio and television.
Question: Do you have a telephone?
Answer: No, I don't.
Question: What means of communication do you think should share health information in your community?
Answer: All of them should share health information. Some people don't have a telephone, but have a radio and so on.

Question: What means of communication do you find more reliable or credible to share health information?
Answer: Doctors share credible health information.
Question: Can you borrow your husband's phone to listen to health information?
Answer: No, it is not possible. In the rainy season, we work in different places. We don’t see each other.
Question: You dial 37321 to listen to the health information. You need to have a minimum balance of CFA 100, but it doesn’t consume your balance in your account. Will you call the service if it is free?
Answer: Yes, I will.
Question: Why will you call it?
Answer: I will call it to benefit from it.
Question: What other ways do you suggest to reach all the community members with health information besides telephone, radio, television, and doctors?
Answer: I suggest doctors at the health centers call for health meetings. Many people will attend them. We don’t have town criers in our community.
Question: Have you ever heard about the Viamo Service?
Answer: No, I haven’t.
Question: Who do you think should give you health information?
Answer: Doctors at the health center.
Question: Can many people use phones to get health information in your community?
Answer: No, they can’t. Only a few people can do it
Question: Why many people can’t use phones to get health information?
Answer: They don’t know about the service.
Question: Do many people have phones here?
Answer: Yes. Many people have phones here.
Question: We want many women to get health information. What do you suggest?
Answer: You need to work hard to inform them about the service.
Question: What to do to encourage husbands and wives to listen to the service together on the phone?
Answer: Men won’t accept to listen to it with their wives.
Question: What about sensitizing them?
Answer: It is not easy, but you can try it.
Question: What special health issues do your community members want to learn more about?
Answer: We want to learn more about malaria and stomach ulcers. We suffer a lot from them here.
Question: What to do to make women listen to the service and get benefits?
Answer: Doctors can sensitize them through talk meetings on health.
Question: How to reach women who don’t attend health centers?
Answer: They can get sensitized during women’s weekly meetings.
Question: What to do to make a group of women listen to the service together on the phone?
Answer: In the rainy seasons, they are busy on the farms. It may be possible in the dry season.
Question: What to do to make a group of women listen to the service together on the phone in the dry season?
Answer: In the dry seasons, you can send them some people to inform them.
Question: Do you have any suggestions to help all the women have access to the service?
Answer: Doctors are essential in the field of health. They may be helpful to do it. In the dry season, some selected people can meet women to inform them about the service.
Question: Do you have any questions?
Answer: No, I don’t.

Question: OK, this is the end of our conversation. Thank you very much for your attention.
Answer: You are welcome.

September 1, 2023

Conversation with a woman using Viamo Service in Ferekoroba, Ouelessebougou

Bougouni-U-I-1

Question: How old are you?
Answer: I am 18
Question: Did you attend school?
Answer: No, I didn’t.
Question: What is your occupation?
Answer: I am a housekeeper.
Question: Do you practice any remunerative activity?
Answer: Yes, I do. I am helping my mother sell some goods.
Question: How many children do you have?
Answer: I don’t have any children.
Question: How do people in your community get health information?
Answer: We mostly get it from the radio and telephone.
Question: What other sources do they get health information from?
Answer: They get health information from Oumou Diarra’s Talk Show.
Question: What source do people trust most in your community? Give the reason.
Answer: People believe in Oumou Diarra’s Talk Show on the telephone because it guides people.
Question: Do people here know much about malaria?
Answer: Yes, they do.
Question: What do they know about malaria? How do we avoid getting malaria?
Answer: I learned from Oumou Diarra’s Talk Show to use mosquito nets to protect against malaria.
Question: Do all your community members have access to health information on time?
Answer: No, they don’t. Some have phones, but others don’t.
Question: Don’t they have radios?
Answer: They have radios.
Question: Then, why they don’t have access to the health information?
Answer: Those who don’t have radio and phones don’t have access to health information here.
Question: Are there groups of people who don’t have access to health information due to distance, network issues, age, or sex, to name a few in your community?
Answer: Many young people don’t get health information because they don’t have phones.
Question: What means of communication are more used to get health information in your community?
Answer: Many people have radio and telephone here.
Question: Do you have a radio and telephone?
Answer: I have a radio. I listen to health information on the radio and telephone.
Question: What means of communication do people trust most in your community? Why?
Answer: People believe in Oumou Diarra’s Talk Show on the telephone and radio because it guides people.
Question: Do you have a telephone?
Answer: No, I don’t. But I listen to the service with my husband’s phone.
Question: It is free to call the service even though you need to have a minimum balance of CFA 100. Will more people call the service if they don’t have to get a CFA 100 balance in their account?

Answer: Yes, I do. Many people will call the service when it is free because some people don't always have money.
Question: What other ways do you suggest to reach all the community members with health information?
Answer: I suggest radio and telephone.
Question: Do you find the information on Oumou Diarra's Talk Show relevant? Could you give some advantages you benefited from it?
Answer: It is relevant information. I have learned many things from its advice. It encourages women to practice remunerative activities. I am listening to her advice.
Question: What about health information?
Answer: I haven't listened to it yet.
Question: Do people understand the information on Studio Tamani?
Answer: Yes. It is easy to understand.
Question: What did you learn from the information on Studio Tamani?
Answer: I have learned a lot about family life.
Question: What about health information?
Answer: I haven't listened to it yet. I listen to Oumou Diarra's Talk Show only.
Question: Will you continue to listen to the information on Studio Tamani?
Answer: Yes, I will because of the pieces of advice.
Question: Are there community members who don't have access to information? Why?
Answer: Yes, there are. Some don't have phones.
Question: Which age group do they belong to?
Answer: They are young people.
Question: What to do so that all the community members can listen to Oumou Diarra's Talk Show and the health information on Studio Tamani and benefit from it?
Answer: I don't have any suggestions.
Question: OK, this is the end of our conversation. Thank you very much for your attention.
Answer: You are welcome.

September 1, 2023

Conversation with a woman using Viamo Service in Ferekoroba, Ouelessebougou

Bougouni-U-I-2

Question: How old are you?
Answer: I am 25
Question: Did you attend school?
Answer: Yes, I did.
Question: What educational level did you reach?
Answer: Grade nine.
Question: What is your occupation?
Answer: I am a housekeeper.
Question: Do you practice any remunerative activity?
Answer: Yes, I do. I sell some articles.
Question: How many children do you have?
Answer: I have got two children.
Question: How do you get health information?
Answer: We mostly get it from the radio and telephone.
Question: What are the most reliable sources of health information according to your community members?
Answer: People believe in radio. Many people have radios.
Question: Do all your community members have access to health information on time?
Answer: Yes, they have access to health information. Radio shares information on health.
Question: Do people here know something about malaria?
Answer: Yes, they do. Recently, we received mosquito nets. We learn how to protect against malaria.
Question: Do you know what to do in case of malaria?
Answer: Yes, I know. We go to health centers when we have money to buy the medicaments. When we don’t have any money, we go to the traditional healers.
Question: Are there groups of people who don’t have access to health information due to distance or network issues, to name a few in your community?
Answer: In the past, the network coverage was poor, but there is an improvement today. Health information is available on phones and radios.
Question: What means of communication is more used to get health information in your community?
Answer: Many people have television here, but only a few watch it for health information. We use the radio a lot for health information.
Question: What means of communication should share health information here according to you?
Answer: I suggest radio. It is the most listened to in this community.
Question: What source do people trust most in your community?
Answer: People believe in radio.
Question: Do you have a phone?
Answer: Yes, I have.
Question: Is it possible to use someone’s phone to listen to the formation in your village?
Answer: No, it is not. People won’t accept to lend their phones because phones are personal.
Question: It is free to call the service even though you need to have a minimum balance of CFA 100. Will more people call the service if they don’t have to get a CFA 100 balance in their account?
Answer: Yes, I do. Many people will call the service when it is free because they don’t have money.

Question: What other ways do you suggest to reach all the community members with health information?
Answer: I suggest radio because many people listen to it. Also, talk meetings can be helpful.
Question: Do you find the information broadcasted by Studio Tamani relevant? If yes, name some advantages you benefited from it.
Answer: It is relevant information. I have learned many things thanks to it. It guides people with good advice concerning the life of couples. I had some problems with my mother-in-law in my family, but the advice from the service helped me overcome the situation.
Question: What do you think about the understanding of the health information by Oumou Diarra on Studio Tamani?
Answer: It is easy to understand. It is in the Bambara language.
Question: What do you remember about the information shared on Studio Tamani?
Answer: I listened to Oumou Diarra’s Talk Show only. It guides people on relevant topics.
Question: Will you continue to listen to the information on Studio Tamani?
Answer: Yes, I am still listening to it.
Question: Do you encourage other people to listen to it?
Answer: Yes. I do because it is relevant.
Question: What is its importance?
Answer: The advice it gives on the life of couples.
Question: I know you listen to Studio Tamani. Have you ever heard of health information on it?
Answer: No, I haven’t. I listen to Oumou Diarra’s Talk Show only.
Question: Did you know that it broadcasts information on health issues?
Answer: No, I didn’t.
Question: Now you know it, will you listen to health information?
Answer: Yes, I will.
Question: Do many people listen to Oumou Diarra’s Talk Show?
Answer: Few people listen to it. Many people don’t have phones.
Question: What to do so that all the community members listen to the health information on Studio Tamani and benefit from it?
Answer: I suggest that you share the health information on the radio. Also, I advise you to call meetings to discuss with the community members on health issues.
Question: Husbands have telephones more than wives**.** What to do to encourage husbands and wives to listen to the service together on the phone?
Answer: Some husbands don’t like to discuss with their wives. You need to sensitize men first.
Question: What specific health topic to discuss during health talk meetings?
Answer: I suggest topics on the life of couples.
Question: What about health information?
Answer: People need more information on malaria because it is popular.
Question: What to do to encourage women to learn about health information?
Answer: I suggest that you share the health information on the radio. Also, I advise you to call meetings to discuss with women on health issues.
Question: Many women can gather and listen to the service together. How encouraging them to listen to health information together?
Answer: You need to sensitize more. I suggest talk meetings on health to sensitize them about it.
Question: You suggested talk meetings on malaria. Who should lead them according to you?
Answer: Doctors can lead them. They are health professionals.
Question: Do you have any questions?
Answer: I am happy to talk with you. Thank you very much! I wish you all the best!

Question: You are welcome! See you!
Answer: See you, too.

September 6, 2023

Conversation with a woman using Viamo Service in Ouelessebougou: Bougouni-U-I-3

Awa Guindo: Hello Madam. I was supposed to meet you in Ouelessebougou for this conversation. But, we will do it on the phone call due to the rainy season. But first, I need your agreement to participate in this conversation.
Woman: I agree to participate in this conversation if it doesn’t take much time.
Awa Guindo: Thank you very much. It won’t take much time.
Awa Guindo: How old are you?
Woman: I am around 28.
Awa Guindo: Did you attend school?
Woman: Yes, I did.
Awa Guindo: Which grade did you reach?
Woman: I got a DEF Degree.
Awa Guindo: What is your occupation?
Woman: I was trading in the past, but I am taking a course for teachers today.
Awa Guindo: How many children do you have?
Woman: I have one child.
Awa Guindo: How did you learn about the Viamo Service?
Woman: I learned it from my husband. He is a listener of Studio Tamani.
Awa Guindo: What is the service number?
Woman: It is 37321.
Awa Guindo: Do you listen to health information on Studio Tamani besides “Baroni”?
Woman: I listen to “Baroni” only.
Awa Guindo: How do people in your community get health information?
Woman: I don’t know much about it, but television shares health information sometimes.
Awa Guindo: What other sources do they get health information from besides television?
Woman: I don’t know.
Awa Guindo: Do you trust the health information you get from television?
Woman: Yes, I do.
Awa Guindo: Why do you trust it?
Woman: Because when you listen to its advice, you benefit.
Awa Guindo: Do you know something about malaria?
Woman: Yes, I do.
Awa Guindo: Do you know what to do in case of malaria?
Woman: Yes, I do. In case of malaria, we take the patient to the health center.
Awa Guindo: What to do to avoid getting malaria?
Woman: To avoid it, we must sleep under mosquito nets and clean dirty water puddles in the neighborhood.
Awa Guindo: What to do in case of malaria?
Woman: We take the patient to the health center.
Awa Guindo: Do women in your community know something about malaria?
Woman: in my family, all the women know something about it. I don’t know about other women.
Awa Guindo: Are people in your community facing challenges in accessing health information?
Woman: Yes, they are.
Awa Guindo: What are the difficulties faced by your community in getting health information?

Women: Television is their only source of health information, but many people don’t have it.
Awa Guindo: Are there groups of people who don’t have access to health information in your community on time?
Woman: No, there aren’t.
Awa Guindo: Why do you think that all your community members have access to health information on time?
Woman: I can’t talk about all the community, but I can say something about my family members.
Awa Guindo: What about your family member? Do they have access to health information on time?
Woman: Yes, they do.
Awa Guindo: Why do you think that all your family members have access to health information on time?
Woman: Because they practice the instructions on health information. They sleep under mosquito nets and clean the compound, etc.
Awa Guindo: What to do to spread health information to all the community members?
Woman: They can attend health centers or listen to Studio Tamani.
Awa Guindo: Which source do you listen to to get health information on radio, television, and telephone?
Woman: Telephone is my source of health information.
Awa Guindo: Why do you listen to it to get health information?
Woman: Because television rarely shares health information.
Awa Guindo: What sources of information are more practical to get health information among television, radio, and telephone?
Woman: Telephone is more practical.
Awa Guindo: Do you trust the health information from Studio Tamani?
Woman: Yes, I do.
Awa Guindo: Why do you trust it?
Woman: Because my husband listens to it.
Awa Guindo: Do many women have phones in your community?
Woman: Yes, they have.
Awa Guindo: Do women having phones listen to” Baroni” in your community?
Woman: Yes. In my family, they listen to it.
Awa Guindo: Do you find the information on Studio Tamani relevant?
Woman: Yes, it is. You learn about the newsreel of places you have never gone to.
Awa Guindo: It is free to call the service even though you must have a minimum balance of CFA 100. It doesn’t consume your balance in your account. Will more people call the service if they don’t have to get a CFA 100 balance in their account?
Woman: Yes, I do. More people will call it.
Awa Guindo: Why do you think that more people will call it?
Woman: I shared the service number with many people. They don’t call the service because of a lack of balance.
Awa Guindo: What other ways do you suggest to reach all the community members with health information besides telephone, radio, television, and doctors?
Woman: I don’t know other ways.
Awa Guindo: Do you find the information on Studio Tamani relevant?
Woman: Yes, I do.
Awa Guindo: Could you give some advantages you benefited from it?
Woman: It is beneficial because you get the news on what is happening in the country. Studio Tamani shares much information about what happens in remote places of the country.

Awa Guindo: What about health information? Did you get advantages from listening to Studio Tamani?

Woman: Yes, I got it. I learned how to avoid getting malaria. We must sleep under mosquito nets.

Awa Guindo: Do people understand the information on Studio Tamani?

Woman: Yes. It is easy to understand because the message is in the Bambara language.

Awa Guindo: What did you appreciate about Studio Tamani besides health information?

Woman: Studio Tamani is working hard. It is available anytime. I feel happy when I listen to Oumou Diarra's Talk Show.

Awa Guindo: Will you continue to listen to other health information on Studio Tamani?

Woman: Yes, I will.

Awa Guindo: What special health issues do you want to learn more about?

Woman: I am interested in family planning issues.

Awa Guindo: what else do you want to listen to besides family planning issues?

Woman: I want to listen to all of them.

Awa Guindo: Have you noticed something on Studio Tamani that doesn't encourage you to listen to it?

Woman: No, I haven't.

Awa Guindo: Do all your family members have access to health information on Studio Tamani?

Woman: Yes, they all have access to it.

Awa Guindo: Why do you think so?

Woman: because they all have phones.

Awa Guindo: Do they know how it works?

Woman: Yes, they do. The service itself guides the listener to make her choice.

Awa Guindo: Who has more phones, men or women?

Woman: All of them have phones here.

Awa Guindo: What to do to encourage husbands and wives to listen to the service together on the same phone when the wives don't have phones?

Woman: Studio Tamani can sensitize women to listen together.

Awa Guindo: What special health topics do you suggest sharing on Studio Tamani?

Woman: I can't answer that question.

Awa Guindo: What health issues are you facing in your community?

Woman: We have sanitation problems.

Awa Guindo: What else do you suggest besides sanitation problems?

Woman: Children's healthcare in rainy season.

Awa Guindo: What to do to encourage women to listen to the talk shows on Studio Tamani and learn more about health information?

Woman: I suggest women listen to them on the telephone. Once you listen to Studio Tamani, you will not stop doing it.

Awa Guindo: What to do to inform women about the availability of talk shows on Studio Tamani?

Woman: Town criers can inform them about it.

Awa Guindo: What to do to encourage women to gather and listen to the service together with the same phone and exchange over health issues.

Woman: I suggest sensitizing women to listen together during Sunday female meetings.

Awa Guindo: What to do to have women listen to the service continuously.

Woman: They won't stop listening to it because it is free.

Awa Guindo: Do you have any suggestions to help Studio Tamani success in your community?

Woman: Studio Tamani is working hard. I like what they are doing. I wish you all the best in it!

Awa Guindo: Do you have any questions?

Woman: No, I don’t.
Awa Guindo: OK, this is the end of our conversation. Thank you very much for your attention.
Woman: You are welcome.

September 8, 2023

Conversation with a woman using Viamo Service in Ouelessebougou: Bougouni-U-I-4

Awa Guindo: Hello. My name is Awa Guindo. I am working for Studio Tamani. I am calling you because you used to call the Viamo Service. I want to talk about 10 to 15 minutes on the service. But first, I need your agreement to participate in this conversation.

Woman: I agree to participate in this conversation. It is a good idea to talk about the service.

Awa Guindo: Thank you very much. Where do you live?

Woman: I live in Tinguele in the area of Ouelessebougou.

Awa Guindo: How long is it from Ouelessebougou?

Woman: It is ten kilometers from Ouelessebougou.

Awa Guindo: How old are you?

Woman: I am around 48.

Awa Guindo: Did you attend school?

Woman: No, I didn’t.

Awa Guindo: What is your occupation?

Woman: I am a housekeeper and farmer.

Awa Guindo: Do you practice any remunerative activity?

Woman: No, I don’t.

Awa Guindo: How many children do you have?

Woman: I have five children who are alive.

Awa Guindo: How do people in your community get health information?

Woman: Town criers inform us about the childhood vaccination campaigns. Doctors also tell us about health issues at the health center. Our female local organizations also share health issues.

Awa Guindo: What other sources do they get health information from?

Woman: Radios and televisions share health information.

Awa Guindo: What source do people trust most in your community? Give the reason.

Woman: People believe in all of them because they share the same information with doctors.

Awa Guindo: Do people here know much about malaria?

Woman: Yes, they do.

Awa Guindo: What do they know about malaria?

Woman: They know you will catch malaria if you don’t sleep under mosquito nets. Dirty water puddles in the neighborhood are also sources of malaria. Some foods also cause it.

Awa Guindo: What to do in case of malaria?

Woman: Doctors give us mosquito nets for malaria prevention. They don’t get happy if someone catches malaria in the community.

Awa Guindo: Should patients go to the health center in case of malaria?

Woman: Yes, they should.

Awa Guindo: Do all your community members access health information on time?

Woman: Yes, they do.

Awa Guindo: Why do you think that all the community members have access to health information on time?

Woman: Because health information is available on the radio, with town criers, and at the local female meetings.

Awa Guindo: What means of communication are more used to get health information in your community?

Woman: Many people use the radio and telephone here. I used to call the service to get health information until I lost the service number.
Awa Guindo: How did you learn about the service?
Woman: During a female group work on a farm, a woman gave it to me.
Awa Guindo: Do you know how that person learned about it?
Woman: No, I don't know.
Awa Guindo: What means of communication are most efficient to share health information with your community?
Woman: It is radio because many people listen to it.
Awa Guindo: Do you have a telephone?
Woman: I have a telephone, but it is not working. I am using my sister's phone now.
Awa Guindo: Do your people accept to lend their phones to those who don't have any to listen to health information?
Woman: No, they don't accept. They all use their phones.
Awa Guindo: It is free to call the service even though you must have a minimum balance of CFA 100.
Woman: Some rumors say that when you call the service, it consumes your balance little by little. I am interested in the information on it. I don't care if it consumes or not.
Awa Guindo: It is free to call the service even though you must have a minimum balance of CFA 100. It doesn't consume your balance in your account. Will more people call the service if they don't have to get a CFA 100 balance in their account?
Woman: Yes, I do. Many people will call the service when it is free because they don't always have money to buy a balance. Because some people can't access the service when they are out of balance three days later, they stop calling it. They think it is consuming.
Awa Guindo: What other ways do you suggest to reach all the community members with health information?
Woman: I suggest town criers besides radio and telephone.
Awa Guindo: Do you find the health information on Studio Tamani relevant? Could you give some advantages you benefited from it?
Woman: Yes. Studio Tamani shares relevant health information. I have learned many things from it. I have learned about the importance of the first breastfeeding on babies and the importance of children's nutrition.
Awa Guindo: Do people understand the information on Studio Tamani?
Woman: Yes, they do. It is easy to understand because the message is in the Bambara language.
Awa Guindo: Could you name some advantages of what you benefited from listening to Studio Tamani?
Woman: I have learned about the importance of sleeping under mosquito nets. I also learned about the importance of the first breastfeeding on babies and the importance of children's nutrition. Oumou Diarra's Talk Show was useful for me. Thanks to it, I am still in my marriage.
Awa Guindo: Do you want to get more health information? If yes, what kind of health information?
Woman: Yes. I want to know more about severe malaria and children's illness.
Awa Guindo: Have you noticed something on Studio Tamani that doesn't encourage you to listen to it?
Woman: I don't like what it shares on female excision.
Awa Guindo: Do all your community members access the health information on time?
Woman: In our compound, my mother-in-law and I listen to it. She is in bed with her newborn baby to listen to it now.
Awa Guindo: Who has more phones, men or women?
Woman: All of them have phones here.

Awa Guindo: What to do to encourage husbands and wives to listen to the service together on the same phone when the wives don’t have phones?
Woman: You need to sensitize men first on the radio about it.
Awa Guindo: What specific health topic to discuss during health talk meetings?
Woman: I suggest topics on childbearing and children's illnesses. Many pregnant women are facing difficulties in delivering babies in our village. Sometimes, they go to Ouelessebougou when the health center here can’t do it.
Awa Guindo: What to do to encourage women and youths to listen to the talk shows on Studio Tamani and learn more about health information?
Woman: I suggest calling for talk meetings to discuss with women on health issues at the health centers. Women participating in the talk meetings will inform the other women in the community.
Awa Guindo: Before we call for talk meetings, what to do to encourage women to listen to health information on Studio Tamani?
Woman: You need to sensitize more women and tell them that the service is free. It doesn’t consume balance.
Awa Guindo: What to do to encourage women to gather and listen to the service together with the same phone and exchange over health issues.
Woman: I suggest sensitizing women during talk meetings on health issues.
Awa Guindo: What to do to have women listen to the service continuously.
Woman: When women learn about the service and get sensitized to listen to the service, they will do it continuously.
Awa Guindo: What do you suggest to help Studio Tamani succeed in your community?
Woman: I advise you to keep it up despite the difficulties. Some will listen to your sensitization, but others won’t. Anyway, keep on working. I wish you all the best!
Awa Guindo: OK, this is the end of our conversation. Thank you very much for your attention.
Woman: You are welcome.

Siby, August 30, 2023

Focus group with the community Agents.

Koulikoro-H-FG-1

Woman: OK. Let’s start. When talking, don’t say your names. I will call you according to the numbers. You will be attributed numbers : numbers 1, number 2, 3, 4, 5, 6, 7, 8,9, and 10. The village chief is number 10. If someone wants to talk, he has to raise his hand and say his number before talking. Are you ready?

What are the sources of information about health in your community?

Number 2: The sources of information about the health of our community are Community Health Centers, television, radio, and telephones.

Number 3: People get information about health from the traditional town criers who use drums to share the information. Also, some people get information from the local relays and radio, and others from television.

Woman: Does someone else want to take the floor and say more?

Number 4: I do share the views of the previous speakers.

Woman: Of all the sources you named, which sources of information do your community believe most?

Number 4: They trust in how the sensitization about the health information is delivered.

Number 1: community people trust information delivered by doctors and village authorities.

Number 5: Community members trust in the piece of advice given to them by doctors.

Number 3: In this rural community, people trust information delivered from health agents directly. Villagers believe more in the health agents than those shared on social networks. Also, they believe in what the town criers share because it comes from the village authorities.

Number 2: villagers trust the health information they get from the Community Health Centers.

Woman: According to you, why do the community members trust the information of the Community Health Centers more than the other sources of information?

Number 4: They believe in the community health center agents because the agents are in contact with them. They know how to sensitize them. It is the health agents who treat them when they get sick. Many villagers are healthy because of them. Health is their job. Radio broadcast information only. They don’t get in touch with villagers. So, villagers cannot believe them more than the health agents.

Number 5: The community members watch television and listen to the radio. But, they believe in the local relays who give them health information.

Number 2: For the villagers to trust a health agent, he needs to have good behavior toward them. A patient would think a health agent is like a living God because they save people from illnesses.

Number 3: People trust doctors because they share information concerning their field. The community believes in doctors because of their qualifications.

Woman: is it difficult for people to get information about health in your community?

Number 4: No, it is not.

Number 3: Yes, it is. It is difficult to get information about health in our community. There are some places in Siby where there is no network and electricity. Accessing them by road is not easy because of the bumpy roads. Sometimes, some patients come to community health centers in an emergency for illnesses they could avoid. When you ask them, they don’t know what to say because they don’t have any notion of health. They could avoid it.

Woman: What to do to help all the community members have access to information about health to protect themselves against illnesses?

Number 2: The government needs to do something to improve the roads between the different villages in the area. Also, the community needs sensitization over health issues. People live in poor conditions of life here. They can't afford transportation fees for even distances of 5 kilometers.
Number 3: Community health agents should benefit from financial and technical means. It is essential to have a good network and roads or community health centers. But, the health agents also need to have some materials like transportation means to reach the community. Then, the health agents will share information about vaccination and sensitize them about health issues.
Woman: Do you also mean community health agents need professional development workshops to empower themselves?
Number 2: Yes, I do. I want community health agents to benefit from financial and technical support. Then, the Government can also see how to build infrastructures for the local communities.
Woman: Are there some groups of people who cannot get access to the information about health in your community?
Number 5: Some people in remote places can't access health information. The poor conditions of the roads do not allow means of transportation to go to those places.
Number 1: we cannot access some villagers when they are busy working on their farms.
Woman: What group of people are currently busy working on farms, men, women, or children?
Number 1: All of them. Sometimes, when a health agent goes to some villages for sensitization, he misses them because all the villagers are busy working on their farms.
Woman: What are the reasons why some people don't attend the community health centers for treatment in your area?
Number 3: Some people don't go to the community health centers early because they first start with traditional healers. When the treatment fails, they go to the community health center.
Number 2: People here cannot afford health issues. Most of them work on farms or mining sites. Some people trust traditional medicine more than modern medicine. Others neglect health issues. But, when the disease worsens, they go to community health centers.
Woman: Have you ever heard about Studio Tamani or something about Oumou Diarra's Talk Show on Studio Tamani?
Number 2: I am a crew member of a WhatsApp group on which some members share information broadcasted by Studio Tamani.
Woman: Am I right to say that you don't know if Studio Tamani broadcast information about health on the telephone?
Numbers: Yes, you are.
Woman: We are sharing information about health with the community via phones, but many people still don't know it. Can you suggest some solutions to help the community get informed about it?
Number 2: when you broadcast information in French on phones, most people can't get information. They are illiterate. Also, to call the service on Studio Tamani, you must pay a CFA 100 balance. Only a few people can afford it.
Woman: I inform you that the health information broadcasted by Studio Tamani into Bambara, Fulani, Tamachek, etc. It is right to say that you need CFA 100 to call the service, but calling the service does not use your balance. Does someone else want to talk?
Number 3: You should negotiate with your mobile phone operator partner to make it free from charges for villagers. They can't afford it. Even if the community members know about the service, I don't think they will accept to pay a balance to call it. You can also negotiate with the mobile phone operator to share information about health during phone calls for one month. Before a recipient picks up a phone call, he can listen to the service advertising for a while. I think in this way, many people will learn about the service.

Number 7: I share the viewpoint of Number 3 about negotiating with the mobile phone operator for advertising the service.
Woman: Do you want to take the floor number 8?
Number 8: No, I don't have anything to add.
Woman: It is almost the end of our conversation. I want to give you the floor to make suggestions or add more.
Number 3: Thanks for talking about this relevant topic.
Woman: You are welcome. I also want to ask you to inform community members about the service at Studio Tamani. It broadcasts much information on a variety of topics concerning the life of the community. They need a CFA 100 balance on their account, but the service is free.

August 29, 2023

Conversation with the midwife of the maternity ward of Kalassa, Siby Koulikoro-H-I-1

Question: Where does the community of Kalassa get their information about health issues?
Answer: We are working with relays that are in charge of sharing information about health to the community.
Question: Do you confirm that the local relays share information about malaria and vaccination campaigns to the community?
Answer: Yes, I do confirm it. When we have information to share with the community, we call meetings. All the local relays attend it to receive the message and share it with their communities.
Question: How does the community get health information apart from the local relays?
Answer: A part from the local relays their community health centers.
Question: How does the community get health information apart from the community health centers and local relays?
Answer: We share health information with them during periodic women's association meetings. Those attending the meetings share them with the others.
Question: Of the sources you cited, which do you trust most?
Answer: I trust the local relays and the community health centers.
Question: Why do you trust them?
Answer: I trust them because when I give information to my local relays, they spread it in the community.
Question: Why do you trust the local relays?
Answer: I trust the local relays because they get information from the health agents.
Question: Is it a challenge for people to get the health information here?
Answer: It used to be a challenge, but not today.
Question: What was the challenge in the past?
Answer: In the past, many people didn’t trust modern medicine. They didn’t listen to doctors’ advice. Today, they trust modern medicine.
Question: According to you, misunderstanding was the cause of why many people rejected doctors’ advice. It was a challenge. What shows you that health information is no longer a problem in this community?
Answer: Many people attend our meetings. When we call for meetings to share health information, they come. Even those who were reluctant in the past come today.
Question: Are people in your community expressing their needs about health information like malaria?
Answer: We answered their questions during meetings, but I don’t remember someone coming here to express his health needs.
Question: Are there some minority groups in your community who cannot get health information?
Answer: Maybe they are not interested in the information, but not because they can’t access it.
Question: I want to know if some people in your community don’t have access to health information.
Answer: During childhood vaccination campaigns, we need to explain more to some families before they accept their children to get vaccinated. Few people are still reluctant. They don’t trust modern medicine.
Question: Despite the health information shared by local relays, health centers, radios, and televisions, are there groups of people here who don’t access it?
Answer: It may be possible.

Question: Do you confirm that some people don’t have access to health information?
Answer: When we send local relays to share the information with the community, they go from family to family to share it.
Question: What are the reasons why some people don’t attend health centers for treatment?
Answer: Some people don’t take their children to the health centers because of poverty. Others would prefer traditional medicine. They believe it.
Question: Did you know about the broadcasting of health information by Studio Tamani?
Answer: I knew about Oumou Diarra’s Talk Show on the radio Studio Tamani but didn’t know the phone service number.
Question: What other ways do you suggest to share health information with all the community members?
Answer: I suggest the radio because many young girls listen to the radio here.
Question: What other ways do you suggest?
Answer: I suggest television and telephone for those who don’t have a radio.
Question: Can someone without a phone borrow it to call the service?
Answer: It is not possible.
Question: Do you have any questions to ask?
Answer: We need to get more health information. People believe in the local relays who share the health information we give them.
Question: Thank you very much for your attention.
Answer: You are welcome!

Conversation with a midwife in Siby: Koulikoro-H-I-2

Question: How old are you?
Answer: I am 27
Question: What is your sex?
Answer: I am a female
Question: What is your educational level?
Answer: I am a University graduate.
Question: What is your occupation?
Answer: I am a midwife
Question: Do you practice another remunerative activity?
Answer: No, I don’t.
Question: How many children do you have? Are they alive?
Answer: I have four children. They are all alive.
Question: I wish them a long life. What is your job at the community health center?
Answer: I am in charge of helping women deliver babies, prenatal healthcare, family planning, and family healthcare.
Question: How do people in your community get health information?
Answer: They get health information from many sources: health agents, radio, town cries, and local relays, to name a few.
Question: What sources do they trust much among the sources you cited?
Answer: I trust local relays.
Question: Why do they trust them?
Answer: They trust them. Local relays get their information from doctors.
Question: Do you find it a challenge for your community members to access health information?
Answer: There are some challenges due to illiteracy. Some illiterates are reluctant.
Question: What are the obstacles preventing the community from getting access to health information?
Answer: Accessing health information is not a challenge, but understanding is. The community members believe in the health information from people they know. They won’t trust a stranger.
Question: Are people in your community expressing their needs for health information?
Answer: No, there aren’t.
Question: What are the groups of people with difficulties accessing health information?
Answer: Men are reluctant about health information. When sharing health information, we have to meet them first to discuss with them before we meet men. They won’t let you talk to women first. Women are more welcoming than men for health information.
Question: Do people in your community listen to the health information on Studio Tamani?
Answer: They listen to Studio Tamani via radio, but I don’t think they call the service. Some of them don’t have phones. Others don’t know about the service.
Question: Is network coverage available in your area?
Answer: It is available in the town, but not in the surrounding villages.
Question: What do you suggest to improve or popularize the service for communities?
Answer: I want you to inform and sensitize women about the service. Many people don’t know about the service.
Question: Excuse me. I want to take your photo.
Answer: OK, no problem.

Question: What do you think about this initiative?
Answer: It is a good one. The service will help doctors to share health information with the community. They can listen to the health information regularly.
Question: this is the end of our conversation. Thank you very much.
Answer: You are welcome.

August 31, 2023

Conversation with a vaccination agent at the community health center of Siby:

Koulikoro-H-I-3

Question: How old are you?
Answer: I am 49
Question: what is your sex?
Answer: I am a male.
Question: Did you attend school?
Answer: Yes, I did.
Question: What grade did you reach?
Answer: I went to Secondary School. Then I studied health at health school later.
Question: What is your occupation?
Answer: I am a vaccination agent. I am a senior health technician.
Question: Do you practice any remunerative activity?
Answer: No, I don't.
Question: How many children do you have?
Answer: I have seven children.
Question: I wish them a long life. What is your role in the community?
Answer: I deal with childhood vaccination (from birth to nine months and from 15 to 23 months). I vaccinate kids at the health center and go from village to village to vaccinate kids there, too.
Question: Do you sensitize villagers on health issues?
Answer: Yes, I do. Before vaccinating kids, we sensitize women about the importance of vaccination.
Question: How do people get health information in your community?
Answer: They get it from health agents, local relays, radio, and telephone. Phones share health information, too.
Question: Of the sources you cited, which one do you trust most?
Answer: I trust the local relays and the community health centers. For example, they trust me as a vaccination agent because they know him. They don't listen to someone they don't know.
Question: Is it a challenge for people to get health information here?
Answer: No, it is not a challenge today. We started to vaccinate kids a long time ago. Now, many women understand the importance of health. Some people may remain reluctant because of outer misinformation on vaccination.
Question: How do people in remote places without network coverage get health information?
Answer: They get information from local relays and vaccination agents who go there for vaccination campaigns
Question: As you said, health information is a challenge in the community. Do you know about some minority groups in your community who cannot get health information because of distance or lack of roads?
Answer: In the rainy seasons, some places are not accessible.
Question: is the rainy season the only challenge?
Answer: Yes, it is.
Question: Are people in your community expressing their needs for specific health information?
Answer: it will be interesting for them to get more health information. We hope to have support for more health information.
Question: What kind of health information support do you need?

Answer: We need health information support that will make the community abandon their old habits on health issues. We want change in our community.
Question: Do you mean communication support for a change of behavior?
Answer: Yes, I do.
Question: Are some minority groups like people with disabilities or displaced people who don't have access to health information?
Answer: Yes, some displaced people are fleeing the crisis in the country. They are isolated when they come here. Sometimes, they come to the community health center when they hear about food distribution. We discuss health issues when they come to the health center for food distribution.
Question: What are the reasons why some people don't attend health centers for treatment on time?
Answer: lack of money is one reason. Some people would prefer traditional healers because they are cheaper than modern medicine.
Question: Have you ever heard about health information on Studio Tamani?
Answer: Yes, I have. I used to listen to it.
Question: Do you think that Studio Tamani is relevant?
Answer: Yes, It is. There is a lot of relevant health information on it.
Question: What have you benefited from it?
Answer: It is a scaffolding to me. I learned more about health information I didn't know from it.
Question: Could you give one example of when you learned something from it?
Answer: I learned how to sensitize women who refuse to vaccinate their kids because of the side effects of vaccinations.
Question: Can poor network coverage remain an obstacle to the health information on Studio Tamani?
Answer: Yes, it can. In some places, there's no network coverage. The mobile phone operators are absent there.
Question: Are there many places with no network coverage here?
Answer: No, there aren't many. It is rare.
Question: How many villages do you go to for vaccination campaigns with no network coverage?
Answer: I know two villages (Dioulafondo and Guéna) with weak network coverage.
Question: What other ways do you suggest to share health information besides telephones?
Answer: I suggest the community radios and town criers. Many people listen to them in some places.
Question: Do you agree to take a photo with me?
Answer: Yes, I do.

September 8, 2023

Conversation with the DTC of the community health center of Siby: Koulikoro-H-I-4

Question: How old are you?
Answer: I am 33
Question: What is your educational level?
Answer: I am a university graduate
Question: What is your job at the community health center?
Answer: I am in charge of coordinating the activities of the community health center in the different units. I am the head of the community health center of Siby.
Question: How many children do you have?
Answer: I have got two children.
Question: I wish them a long life. Could you explain your duties and responsibilities in the community?
Answer: I have many duties and responsibilities. I mainly work on sensitization to avoid diseases. As the saying goes: " prevention is better than cure". I ensure that people get vaccinated in my area. The community health center informs women about the importance of prenatal health care.
Question: What else are you doing besides sensitization?
Answer: We heal patients. We help pregnant women to deliver their babies. We both heal patients and sensitize the community on health issues.
Question: What are the principal sources of health information accessible to your community?
Answer: There are community relays, community health centers, doctors, radios, television, and social networks.
Question: What sources do your community people trust much?
Answer: They must trust the health information of the community health centers. But, they also trust other sources like fake news on social media on health information. That's why some people reject vaccination.
Question: Why does the community believe more in the doctors' information?
Answer: There is a good understanding between doctors and the community. People believe in doctors because of their good behavior toward the community.
Question: Is it a challenge for people to get information about health in your community?
Answer: Yes, it is. Doctors don't have access to all the community members. When you call for a sensitization meeting, only a few people attend it. But radio, television, and social media can reach a large audience.
Question: What to do to give health information to a large audience?
Answer: Television, radio, and social media can have access to a large audience.
Question: According to you, what are the obstacles linked to health access in your community?
Answer: One of the obstacles is network coverage. There are some villages in my area with weak networks. Second, the fake news on social media is also an obstacle. Some people believe in the fake news of social media. Some husbands don't support their wives in getting health information. Lack of money is another obstacle.
Question: Could you give an example of when a husband is an obstacle for his wife to access health information?
Answer: Some husbands don't want their wives to listen to family planning information because it is taboo. They think that family planning is against the teaching of religions.
Question: Are there other obstacles besides family planning?
Answer: Religion is also an obstacle.

Question: How can religion be an obstacle?
Answer: Some religious leaders have different views about family planning. They think that family planning means no children.
Question: Are people in your community expressing their needs for health information? If yes, what are they?
Answer: they need network coverage to get health information via telephones. Community health agents can't access all of them. They need to get information without spending money and staying in their locality.
Question: How do your community members want to access health information besides radio, television, and telephone?
Answer: As I said, they would prefer the community health agents and local relays. They meet them and discuss health topics.
Question: What is the most known group of people having difficulties accessing health information?
Answer: They are teenagers and young people. It is difficult to reach them.
Question: Why is it difficult to reach them?
Answer: They don't have the financial autonomy to have a radio, telephone, or television.
Question: What are the reasons why some people don't attend health centers for treatment on time?
Answer: Some rumors on social media and some influential people giving wrong information to the community members.
Question: How do some influential people impact the community?
Answer: They frighten them, telling them they will die or get ill if they do this treatment.
Question: Have you learned about Viamo Service or Studio Tamani?
Answer: I have learned about Studio Tamani, but not Viamo Service.
Question: Do you listen to Studio Tamani?
Answer: Yes, I do.
Question: How do you listen to Studio Tamani?
Answer: I listen to it on social networks.
Question: The Viamo Service contains a lot of health information. You can always listen to them. it is free to call the service when your account is active. Will many people call the service if they don't need to have an active account?
Answer: Yes. Many people will listen to it, but you must inform them about the service.
Question: Why will people many people listen to with a non-active account?
Answer: The health information on Viamo Service is essential for people in remote places who don't have access to information. In those places, people live in poor conditions. They can't afford a phone balance. They can't have access to balance In some villages.
Question: Is the network coverage available for all the community members?
Answer: During one of our mosquito net distribution in a village, we noticed that few people have phones in families. In another, they had phones with no network coverage.
Question: What group of people don't have phones, women, men, children, or displaced people?
Answer: it is women. Many of them are illiterate.
Question: What do you suggest Viamo Service do to be accessible to all the communities?
Answer: You can call meetings and discuss the importance of health information.
Question: What do you suggest besides the Viamo Service to access health information, especially for women on time?
Answer: You can empower health information on radio and television.
Question: What do you suggest to do to convince men to listen to the service with their wives?
Answer: You can sensitize them from village to village about the importance of the service.

Question: Who do the men of the community trust to conduct sensitization programs in the community?

Answer: In my area, they will trust a health caravan going from village to village to sensitize them. Local relays, doctors, and local authority members should be part of the health caravan.

Question: What health information is essential to be part of the sensitization?

Answer: I love his question a lot. We want people to get interested in Childhood vaccination. Also, women should learn about family planning and prenatal health care.

Question: How to promote the Viamo service for women and family members?

Answer: I suggested a health caravan. Men, women, and children will learn with the health caravan.

Question: What do you suggest to do to help many people attend the talk meetings and exchange about health issues?

Answer: In my place, it is easy. When town criers share information about talk meetings, people will attend.

Question: What do you suggest to do to continue the talk meetings at the end of the project?

Answer: A committee can volunteer for the follow-up of the project.

Question: What do you suggest Viamo Service to improve for the well-being of the communities?

Answer: I suggest that you consider the needs of my community into account.Thanks, Viamo Service, including its heads and all the staff members.

Question: Are you talking about the needs you stated earlier?

Answer: Yes.

Question: I will share them with the Viamo Responsible. Thank you for your attention

Answer: You are welcome.

Siby, August 30, 2023
One-to-one conversation with a midwife in Siby
Koulikoro-H-I-5

Question: How old are you?
Answer: I am 27
Question: What is your sex?
Answer: I am a female
Question: What is your educational level?
Answer: I am a University graduate. I have got a license.
Question: What is your job at the community health center?
Answer: I am in charge of helping women deliver babies, prenatal healthcare, and family planning.
Question: Do you practice any additional remunerative activity?
Answer: No, I don’t.
Question: How many children do you have?
Answer: I have got three children.
Question: What are the sources of health information in your community?
Answer: Radio and television are sources of health information in the community. When doctors share health information with us, we share it with people attending the community health center. For example: On Tuesdays, they go to the health center to learn about malnutrition. Local relays share health information with their community, too.
Question: What sources do you trust much among the sources you cited?
Answer: I trust the national television station.
Question: Why do you trust television most?
Answer: I trust all health information. I trust NGOs working in the field of health, too. We work in partnership with some NGOs.
Question: Why do you trust them?
Answer: I trust all of them, but mostly television.
Question: Are there any challenges for people to get the information about health in your village on time?
Answer: Yes, there are some. We are in a rural community where sensitization is difficult. We need to be patient in sensitizing the community members.
Question: What are the reasons why some people don’t access to health information?
Answer: In rainy seasons, people work on farms. They are not available for health issues.
Question: What other difficulties are you facing in sharing health information with your community?
Answer: There isn’t any, apart from the rainy season period.
Question: Are people in your community expressing their needs for health information?
Answer: Yes, there are some.
Question: How do people in your community want to get health information?
Answer: On market days and Wednesdays (malnutrition day at the health center) many women from the surrounding villages come here. Some seek health information from us.
Question: Do community members seeking health information go to the community health center?
Answer: Yes, they do.
Question: Do they use other sources for health information?
Answer: Yes, they do. They listen to the radio to get health information.
Question: What are the groups of people with difficulties accessing health information?

Answer: The groups of people having difficulties accessing health information are elders who stay at home.
Question: Why elders don’t get health information?
Answer: They stay in. They don’t have radio and television. Only a few of them have a cellular phone to listen to information.
Question: What are the reasons why some people don’t attend health centers for treatment on time?
Answer: Many people here come to the health center for treatment late. They come when their illness is at a dangerous level. When you ask them, they say they don’t have money to buy the medicines.
Question: Do you know other reasons why some people don’t attend health centers for treatment on time besides lack of money?
Answer: Some people are illiterates. They don’t believe in modern medicine. They would prefer traditional medicine.
Question: Do you find it relevant to broadcast health information in your community?
Answer: Yes, it is. It will be helpful for our community. I gave the service number to many people here and in Bamako. I will give it to many people. I want everybody to access health information.
Question: Do many people have phones in your community?
Answer: Yes, they do. Lots of people have phones in my community.
Question: What do you suggest to improve or popularize the service for communities?
Answer: I want you to work hard to inform people about the service. When the service gets popularised, people will get health information while staying at home.
Question: What means of information do you suggest besides the telephone to give health information to women?
Answer: I suggest vaccination agents. They meet many women during vaccination campaigns. Women believe in their information and participate actively in vaccination and malnutrition campaigns. Female leaders can also share health information with the members of their organizations.
Question: Do you have any questions to ask me?
Answer: No, I don’t have any. I want to thank you for coming here.

Siby, August 29, 2023

Conversation with the village chief and his councillors: Koulikoro-L-FG-1

Woman: OK. Let's start. When talking, don't say your names. I will call you according to the numbers. You will be attributed numbers like 1, number 2, 3, 4, 5, 6, 7, 8,9, and 10. The chief is number 10. If someone wants to talk, he raises a hand and says his number before talking. Are you ready?

Man: What are we going to talk about?

Woman: We are going to talk about the information broadcasting by telephones. Question 1: How do you get health information?

Number 7: We get most of our information about health from the Community Health Center.

Woman: We want to have more people answer the same question. If you also want to confirm a statement, you take the floor. But if someone has different answers, he can take the floor and give his answer.

Number 3: I confirm the answer Number 7mber7. We get information from our Community Health Center.

Woman: Who else wants to take the floor and say more?

Number 4: I confirm the answer given by Number 7.

Woman: Who else wants to say something about question 1?

Number 1: I also confirm what the previous numbers say.

Woman: A part from the Community Health Center, where do you get information about health issues?

Number 7: We get health information from local relays, community radio, and television, too.

Woman: OK, thank you. Someone to speak?

Number 3: Yes, I agree with Number 7.

Woman: who else wants to add something?

Number 55: I confirm, too.

Woman: As you said, you get information from the local relays, the Community Health Center, Community radio, and television. Which source do you trust more?

Number 3: We trust the information shared by our local relays via community radios.

Number 4: I confirm what Number 3 said.

Woman: Does someone else want to talk?

Number 2: we trust the community radios. We get a lot of information from them.

Number 1: I trust information broadcasted by radio and television.

Woman: who wants to take the floor?

Number 7: We also get information from the town criers.

Woman: so, you said that when you get information from the local relays, the Community Health Center, Community radio, television, and the town criers, you trust them. Why do you trust them?

Number 7: We trust the workers in charge of sharing the information.

Number 3: I share the view of the Number 7.

Woman: who wants to add something?

Number 5: We trust the workers in charge of sharing the information.

Woman: Does someone want to talk?

Village chief: The local relays in charge of information via radio get it from doctors. This is the reason why we trust it.

Woman: What else do you want to say?

Number 4 and 2: We agree with what the village chief said.

Woman: Are you facing challenges to get health information in your area?
Number 10: Doctors provide information about malaria, prenatal care, and children's health. We trust in everything doctors say about health, but we don’t trust in unknown sources of information.
Woman: This is the Number 10’s answer. Do you want to add something?
Number 5: The village chief is right.
Number 6: I also share the view of the village chief.
Number 1 and 2: We do share his point of view.
Number 8: We are not facing challenges getting health information here.
Woman: Is it correct to say it is easy for people here to get health information?
Number 7: Yes, you are.
Woman: You all said you trust the information you get about health. Do you know people in your village who don’t trust them?
Number 7: Yes, some people don’t trust that information.
Woman: What group of people do you think don’t trust that information? Are they women, men, young people, or displaced people?
Number 7: Some chiefs of families don’t trust the health information.
Woman: Are they males or females?
Number 7: They are males.
Woman: Does someone want to say something?
Number 1: Yes, I agree with Number 7.
Woman: What is the reason why they don’t trust the information?
Number 7: They don’t trust the information because some social networks broadcast fake news.
Woman: Can you explain it? I don’t get you well.
Number 7: Some social networks broadcast information that discredits what the local relays say.
Woman: Can you give one example of when social networks discredit the information of the local relays?
Number 7: I cannot name precisely a social network, but this is what some people say here.
Woman: Are the discredits about health information?
Number 7: Yes, they are.
Woman: Does anyone want to answer the question? What group of people do you think don’t trust that information?
Number 3: I agree with Number 7. Some groups of people rebel against the information shared by local relays. They discourage people from using some medicaments suggested by local relays.
Number 8: It is due to a misunderstanding. Sometimes, when a vaccinated person gets ill, some may think it is due to the vaccine. This is one of the reasons.
Woman: Does someone want to take the floor?
Number 4: I do agree with what my colleagues said.
Woman: Many people get ill and collapse, but some refuse to go to the hospital early despite the large amount of information broadcasted about health issues. Could you tell me why some people don’t go to the hospital for treatment?
Number 10: People want to go to the health Center, but it is far from the village.
Number 7: One of the reasons why people don’t attend hospitals is the lack of money.
Woman: OK, to sum up, you think sick people don’t attend health centers because of lack of money and the long distance between the village and the health center? Do you want to add something?
Number 6: I share the same answer with Number 7. In some families, people can’t afford the expenses of health. The health center is far from the village. They have to pay for transportation fees. Lack of money is the main challenge because everything depends on it.
Woman: OK, do you want to add more?

Number 3 and number 5: We do agree with them. Money is the main reason.

Woman: Studio Tamani broadcasts much information. You can call the service for free on condition that you have a balance of CFA 100 in your phone account. For those who used to call the service, do you find relevant the information broadcasted?

Number 7: All the broadcasted information for sensitizing people is relevant. I used to call the service from time to time.

Woman: Number 7 says that the information is relevant. What did you benefit from it?

Number 7: I benefited from health and family education a lot.

Woman: Now, number 5 has the floor.

Number 5: The service is relevant because one can listen to the newsreel without a radio set.

Woman: Who wants to take the floor?

Number 6: I share the point of view of Number 5.

Number 1: I want to add something. The service improves one’s knowledge. One can learn something by calling the service.

Woman: Has the information broadcasted by the service 37321 brought changes to the community of Siby? Do people go to the hospital, or do kids sleep in mosquito nets because of the sensitization of the service? Does the rate of vaccination increase because of the service, or does women's delivery in the health center increase? What changes did you notice as an impact of the service?

Number 10: People have learned a lot about the importance of attending health centers. More and more people attend health centers because of the service.

Woman: Someone else?

Number 7: Because of the service, people are more interested in prenatal care today.

Woman: Does someone want to add more about the changes brought by the information of the service?

Number 5: Women listen to the service, learn more about health information and family planning, and then share with their husbands for decision-making. Women find Oumou Diarra’s talk show about family issues helpful for family life.

Women: Who wants to take the floor?

Number 7: Kunafoninw increased sanitation in our area. Many people use soap to wash their hands.

Women: To call the service, you must have a phone and a network. Are you facing network issues to call?

Number 7: We are not facing any network issues here in Siby.

Woman: Do you want to add something?

Number 3: We don’t have a network issue here.

Woman: If you are not facing network issues, why are many people not listening to the service?

Number 3: The reason is due to a misunderstanding. Some people don’t know about the existence of the service.

Woman: Does someone want to add something? Why are many people not listening to the service?

Number 7: I do agree with Number 3.

Woman: So, you think they haven’t learned about the service yet?

Number 7: Yes, I do.

Woman: Who wants to say something?

Number 10: Some people haven’t learned about the service, but young people are getting more and more interested in the service.

Woman: Number 1, do you want to talk?

Number 1: Those who don’t listen to the service haven’t learned about it yet.

Woman: What to do to help people learn about the service in Siby?

Number 10: Phone and radio listeners are interested in childishness and less relevant things.

Woman: What to do to encourage people to learn about the service in Siby?
Number 10: We can face this situation by thinking about it.
Woman: Number 7: You have the floor.
Number 7: We need widespread news about the service.
Number 3: We can talk about the importance of the service to people.
Woman: who wants to take the floor?
Number: You should broadcast the information about the service on television because some people don’t have phones. In this way, more people can get access.
Woman: Now, what to do, especially for females to help them learn more about family issues like kids' healthcare and family healthcare?
Number 1: Many women don’t have phones. We need to broadcast the information about the service on radios and televisions if we want more women to get health information.
Woman: Who else wants to talk?
Number 8: Women are more interested in entertainment and amusement activities. You can attract them by organizing amusement activities integrating health information. They are more interested in Oumou Diarra’s Show talks for this reason.
Woman: Yes, do you want to talk?
Number 3: Yes. I do share his opinion. Entertainment or amusement when broadcasting the information about the service can help draw the attention of many women.
Woman: Do you all have phones? How many of you don’t have a phone?
Counselors: Two people don’t have a phone.
Woman: OK, two out of ten counselors don’t have a phone. If you don’t have a phone, can you borrow someone else’s phone and listen to the service?
Number 1: We can listen to the service with our phone, but we cannot use someone else’s phone. we don’t have time for it.
Woman: Who else wants to talk?
Number 3: I agree with Number 1, Madam. You cannot borrow somebody’s phone to listen to the service.
Woman: Number 4. You have the floor.
Number 4: I do share their viewpoint.
Woman: What about women? Can they use somebody’s phone to listen to the service?
Counselors: No, they can't.
Woman: Now I want to give you the floor. We are at the end of our conversation. Do you have any suggestions to make?
Number 3: During our conversation, we talked about relevant issues. I suggest you give information about the service to people who haven’t learned about it yet.
Woman: Who wants to add something else?
Number 6: as number 3 said, you need to spread the information about the service so that many people can learn about it. Meet them and talk to them like you are doing it now. When ten people know about it, they will inform many people.
Woman: One more question before we end this conversation. You need CFA 100 in your balance account to call the service even though it doesn’t use your balance in your account. Do you think many people will be able to listen to the service if they are allowed to do it without a CFA 100 balance?
Number 10: I don’t think the CFA 100 is a challenge for people here. You only need to spread the news of the service to a large audience.
Number 2: I want you to give me the service number. I want to register it on my phone and call the service.
Woman: OK, I will give you the service number after the conversation. Number 1, you have the floor.

Number 1: I would love to listen to the service unfortunately, I don’t have a phone.
Woman: you have the floor number 6
Number 6: Since some people don’t have a phone, why don’t you use different ways to provide information on the service to people?
Woman: What other ways could you suggest?
Number 6: I suggest radio or television. Those who don’t have a phone can go and watch television or listen to the radio to people possessing them. Telephones are personal devices, but radio and television are not in Siby.
Woman: who wants to add something?
Number 7: I see eyes to eyes with him.
Woman: what else do you want to say?
Number 8: what is your project about?
Woman: OK, I will answer this question after the conversation. This is the end of our conversation. Thank you very much for your attention. I do apologize if I did anything wrong during this conversation.

August 30, 2023

Conversation with the community authorities in Siby: Koulikoro-L-FG-2

Woman: Let's start. Please speak aloud when you have the floor. Question 1: How do you get the information about health in your area?

Number 4: I get the information about health from the Community Health Center.

Woman: Do you want to take the floor?

Number 2: I get information about health from the local relays.

Number 1: I get information about health from the Community Health Center and the local relays.

Number 8: As a local relay member, we get the information from the Community Health Center, radio, television, and telephone.

Woman: As you said, you get information from the local relays, the Community Health Center, Community radio, and television. Which source do you trust more?

Number 7: We trust the information from the Community Health Center.

Number 4: We trust the information from the Community Health Center. They give us relevant health information.

Number 10: We trust the information broadcasted by television and radio.

Woman: Besides your local Community Health doctors, you trust television and radio even if you don't know those who state them.

Number 10: Yes. I trust them because they broadcast to a large audience. I don't think they can lie to them.

Woman: Does someone else want to talk?

Number 3: We trust our local leaders in charge of health. We trust all the information about health they share with us.

Woman: As community authorities, do some people in your area come and see you to seek health information?

Number 3: Yes, some people come and seek information about health from me. As a member of the local relay, I teach them what I know. If I don't know something, I ask my superiors for help.

Woman: Can you give me one example of someone seeking information about health from you?

Number 3: Some people seek information about malnutrition from me.

Number 7: Almost ten persons sought information about malaria from me. A married couple came to see me to learn about family planning four days ago.

Number 8: Some seek information on family planning from me, too.

Woman: When they seek health information from you, do you answer all of their questions?

Number 7: Yes. They get back satisfied with the answers I give them.

Woman: Are people in Siby facing challenges to get health information?

Number 3: Local radios in Siby broadcast health information. Local relays meet people and share the same information with them, too.

Number 4: It is difficult to get information about health in Siby. Both local radios and local relays share with people. Those who don't have access to the radio can meet the local relays for the same information.

Number 2: I do agree with them. People have alternatives here: local radios or local relays.

Woman: Are there groups of people in your area who don't have access to information about health due to the distance, a river to cross, or a mountain to climb?

Number 4: All the local relays collaborate to share information with all the community members. Everyone has access to the information about health in our community.

Woman: According to four everybody has access to information about health.
Number 3: Our local relay is about more than 30 persons. Our leader is number 7. He shares all the health information he gets from the head doctor. Then, local members share the same information with their village people. In some places, there is no radio cover. Local relays become their source of information.
Number 8: I want to add more. As number 3 said, villagers selected some people to work with the Community Health Center. They share with their communities what they learn from the doctors of the health centers. Sometimes, in rainy seasons, farmers are busy working, therefore they are not accessible.
Woman: What to do to reach people during rainy seasons?
Number 8: During the rainy season, improve the network in place with a network problem. Also, provide villages with radio stations.
Number 4: We should receive means of transportation to reach people with a network issue.
Woman: As you said, the community gets information via telephone, Community radio, and television. Which source do your community members use a lot?
Number 7: Television, radio, town criers, and local relays are the sources. Telephone shares information in the French Language. Many people don’t understand it.
Number 8: People are interested in the information of the local relays because they speak in the local language. Telephones broadcast information about health in French, which many people don’t understand. Television broadcasts most of the information in French. Only a little is in the Bambara language.
Number 4: Few people listen to the radio. Local relays remain the most used source in the community. As soon as the relay members get information from the doctors of the community health center, they share it. For example, today is a vaccination campaign day. All the families got the news last night about it.
Woman: So, are the relays the principal source of information for the community?
Number 4: Yes, they are.
Number 3: Local relays share information they get with all the community members. Few people listen to the radio. Few people have television sets, too. So, local relays remain the principal source of information for the community.
Number 10: We villagers trust our local communities.
Woman: Do you all possess a phone?
Number 1: I don’t have a phone.
Woman: Can you borrow a phone from someone to listen to Oumou Diarra's talk show?
Number 1: I don’t listen to Oumou Diarra’s Talk Show.
Woman: Can people in your community who don’t have a phone borrow other people’s phones to listen to the information about health?
Number 7: Phones are personal devices. People don’t share their phones with other people here.
Woman: You need CFA 100 in your balance account to call the service even though it doesn’t use your balance in your account. Do you think many people will be able to listen to the service if they are allowed to do it without a CFA 100 balance?
Number 4: Yes, if people can call the service without a CFA 100 balance in their account, many people will call the service. Listeners will increase when they can call the service without it.
Number 8: I used to call the service to listen to information. But, without a minimum balance of CFA 100, I couldn’t have access to it. That's the reason why I stopped to call the service.
Woman: Has the information about health brought changes in your community? Was it relevant?

Number 8: I can confirm that the information was relevant to our community. Relays share the health information they learned from the community health center. When they don't know something, they ask the doctors at the health center.
Number 4: I confirm what Number 8 said. People believe in local relay members. When they don't know something, they ask the doctors at the health center.
Number 7: I confirm that health information brought changes in our community. Every year, I go to Kati for the assessment. Many people attend the community health centers. People are more and more interested in prenatal care and vaccination.
Woman: OK, there is an increase in prenatal health care, vaccination, etc.… What do you think about the information broadcast by Studio Tamani as Oumou Diarra's Talk Show?
Number 7: I suggest spreading information on the service of Studio Tamani. Some people don't know what it is. How can one listen to something you don't know its existence?
Woman: Are you facing a network issue when calling the service in your area?
Number 7: it is difficult for them to call the service in places with weak networks.
Number 8: Some villages in our area have no access to the network. They cannot access the service. They also don't have radio coverage.
Number 7: I want to add something to what number 8 said. There is a village called Guena. When you want to call them, you call someone in another village 7km from Guena who gives them the information.
Number 4: In my village, it is difficult to access the service. You need to move from one place to another in search of a network. We are 7 kilometers from Siby.
Woman: We use different ways to share information about health with the community. Can you suggest other helpful ways to have access to people in remote places or those with network or radio coverage issues?
Number 7: People who broadcast health information should be motivated to do it.
Number 3: I have been working as a local relay member for about 20 years. I started with many people who stopped serving the community because they said they were unpaid. I accepted to work as a relay to learn more about health issues. Whether I get some money or not, I will benefit from the knowledge I gain. Recently we were given some bicycles. As number 7 said, you need to motivate us.
Number 8: Local relays should be encouraged and motivated with some money. It is difficult for local relay members to walk from one place to another. They need a means of transportation. With a means of transportation, they can go far and spread information about health to communities.
Woman: Let's talk about women. They perform many activities. They go to the market and sell goods and work on farms. They are always busy. What to do to help them access the health information to take care of their family health care?
Number 7: Women like entertainment and amusement. If you organize meetings to entertain them, it will be easier to share health information with them. It can also be a talk show broadcast on radio.
Number 3: Many women are working on farms at this moment. They will love it if someone joins them to chat about health information.
Woman: It is almost the end of our conversation. I want to give you the floor to make suggestions or add more.
Number 4: I want to thank you for everything you are doing. I thank all those who agreed to attend this meeting.
Number 10: I want to thank all of you, too. I wish you all the best!
Woman: You are welcome. That's the end of our conversation. Thank you very much for your attention. I do apologize if I did anything wrong during this conversation. See you next time.

Siby, August 30, 2023

Group discussion with women who don’t use Viamo.

Koulikoro-NU-FG-1

Woman: How do you get the information about health in your area?

Number 1: We get health information from television and radio.

Number 10: She is right. We get information about health from the telephone, too.

Number 8: We get information about health from the telephone, radio and television. We like to listen to it a lot.

Woman: where are we talking at the moment?

Numbers: We are at the community health center.

Woman: Do you get the information about health from this health center?

Numbers: Yes, we do.

Woman: So, you get information about health from your Community Health Center, television, radio, and telephone.

Numbers: Yes, that’s right.

Woman: Of the sources you cited, which one do you trust more? Why?

Number 8: I trust television, radio, and the Community Health Centers because they give us more information on vaccination and the use of mosquito nets.

Woman: You get information on malaria and family health care from television, radio, telephone, and your health center. Is this information enough for you to take care of your family?

Number 8: Though the information we get about health is enough, we want them to add more.

Number 10: Health is relevant. Health centers help us understand how to prevent malaria. We want the sources of information to add more health information.

Number 5: It is enough, but we need more information to face health issues.

Number 10: I like how you are working hard to help our community learn about health issues.

Woman: Do you face any challenges in getting information about health in your community?

Number 10: Only a few people have a phone or a television to get information about health in this community.

Number 8: Few people have a television. One of the challenges we are also facing is power shutdown. Without power, we cannot use our televisions.

Number 4: some people don’t have a phone or a television to get the information. People listen to the radio from time to time.

Number 3: In my area, people have phones, radio, and television. We don’t face any problems getting health information.

Number 6: People don’t have radio and television to get the information unless they go to the community health center.

Woman: Do people in your community get health information?

Number 6: Many people don’t have a television at home. They need to go to watch it with their neighbors. It is not easy for them to do it frequently. Sometimes, to get information, you need to go to another village.

Woman: What to do to overcome the challenges to get health information?

Number 6: People need help to get health information.

Woman: What kind of help do they need? Do they need electricity, or do they want more advertisements for health information?

Number 6: They need electricity. There is electricity in the town, but only a few people can afford it. Some people use solar panels instead. During sunny days, they have more power, but on the contrary, they don’t have the energy to use their devices.
Number 3: I suggest more spreading of the health information.
Woman: As you said, you get information from television, radio, and telephone. Of all these devices, which one do you have?
Number 10: I have a television and a phone, but I don’t have a radio set.
Number 2: I have a telephone and a television. I get information from them.
Number 1: I don’t have a telephone or a television.
Woman: Is it possible to use your husband’s phone to get health information if you don’t have one?
Number 1: No, it is not possible.
Number 4: I don’t have a television, a telephone, or a radio.
Woman: Can you borrow someone’s phone to get information?
Number 4: No, I can't.
Number 5: I am not allowed to use my husband’s phone.
Number 6: I don’t have a phone or a television. I use the radio to get information.
Number 8: I don’t have a telephone. I have a radio set. I use my husband’s phone to get information from time to time.
Number 9: I have a phone, unfortunately, I don't have enough time to listen to information. I am busy nursing my kids.
Number 10: I have a phone and a TV set. I use them to get information.
Woman: Of phone, TV, and radio set, which to use to broadcast the health information?
Number 8: Television is more efficient in doing it.
Woman: Do you trust information when they are on TV?
Number 8: Yes, I do.
Number 4: I do trust TV.
Number 10: I do trust TV, too.
Number 1: No, I don't. I would trust the information given by the community health centers.
Woman: Now you have learned about the service. Are you going to call it if it is free?
Number 4: Yes.
Number 1: Yes.
Number 2: Yes.
Number 5: Yes.
Number 6: Yes.
Number 7: No, because I don’t have a television, a radio set, or a phone.
Number 8: Yes, I will call it.
Number 9: Yes, I will, too.
Woman: Besides telephone, radio, and television, what other ways do you suggest to get health information?
Number 8: We want local relays to come to our villagers and inform people about health.
Number 1: I suggest this kind of conversation about health in our area.
Woman: This is a large country. We can’t go everywhere. It is not easy for us to meet you and talk about health reasons why we use the telephone to broadcast health information regularly. What ways do you suggest to share the health information with community members?
Numbers: Telephone is a relevant means of communication, but only a few people have it.
Woman: Did you learn about the service broadcasting health information on the telephone?
Number 4: I didn’t know it.
Number 5: I didn’t.

Number 8: I didn’t.
Number 6: Me, too, but I didn’t have a phone.
Number 9: I didn’t know it.
Number 10: I didn’t.
Woman: Who used to call the service?
Number: I would listen to the service with my husband’s phone.
Woman: You knew about him, but did you listen to the information about health?
Number: No, I didn’t. I listened to Oumou Diarra’s Talk Show only.
Number: I knew about it when I didn't have a phone. I listen to Oumou Diarra’s Talk Show on the radio.
Woman: Those who didn’t know about the service, who you think should tell you about it.
Number: People in the neighborhood could tell me about the service.
Number 4: My husband should have informed me because he had a radio and a telephone.
Number 8: I think my husband should have informed me
Number 6: I knew about it when I haven’t a phone to call the service.
Number 10: Television should have done it.
Woman: Is the service available for everyone in your area?
Number 4: I don’t think everybody can have access to the service in our community. People possessing a phone can listen to it, but those who don’t have a phone can’t.
Number 2: Not everybody can access the service because they don’t have a phone.
Number 1: Not everybody can access the service because they don’t have a phone.
Number 6: Many people cannot access the service because they don’t have a phone.
Number 8: Many people can’t access the service.
Number 5: Not everybody can access the service because they don’t have a phone.
Number 7: Many people can’t access the service.
Number 9: I have many household chores to do. I don’t have time to listen to the service.
Number 10: Many people cannot access the service because they don’t have a phone.
Woman: What do you suggest to improve our job?
Number 1: You are working hard. I encourage you to continue to work hard.
Number 2: I also encourage you to keep it up.
Number 7: I suggest you inform people about the service.
Number 5: You should do more to reach many people.
Number 10: I like the way you are working. I suggest meeting us from time to time to talk about health issues.
Number 9: What you are doing is helpful for us. I want you to continue with this great job.
Number 6: I like the way you are working. I suggest you to do more.
Number 8: I want you to do more. I like what you are doing. I wish you all the best!
